# Do stressful conditions make adaptation difficult? Guppies in the oil-polluted environments of southern Trinidad

**DOI:** 10.1111/eva.12289

**Published:** 2015-09-04

**Authors:** Gregor Rolshausen, Dawn A T Phillip, Denise M Beckles, Ali Akbari, Subhasis Ghoshal, Patrick B Hamilton, Charles R Tyler, Alan G Scarlett, Indar Ramnarine, Paul Bentzen, Andrew P Hendry

**Affiliations:** 1 Redpath Museum and Department of Biology, McGill UniversityMontreal, QC, Canada; 2 Department of Life Sciences, The University of the West IndiesSt. Augustine, Trinidad and Tobago; 3 Department of Chemistry, The University of the West IndiesSt. Augustine, Trinidad and Tobago; 4 Department of Civil Engineering and Applied Mechanics, McGill UniversityMontreal, QC, Canada; 5 School of Biosciences, University of ExeterExeter, UK; 6 Biochemistry Research Center, University of PlymouthDrake Circus, Plymouth, UK; 7 Department of Biology, Dalhousie UniversityHalifax, NS, Canada

**Keywords:** adaptation, ecotoxicology, habitat degradation, natural selection and contemporary evolution

## Abstract

The ability of populations to rapidly adapt to new environments will determine their future in an increasingly human-modified world. Although meta-analyses do frequently uncover signatures of local adaptation, they also reveal many exceptions. We suggest that particular constraints on local adaptation might arise when organisms are exposed to novel stressors, such as anthropogenic pollution. To inform this possibility, we studied the extent to which guppies (*Poecilia reticulata*) show local adaptation to oil pollution in southern Trinidad. Neutral genetic markers revealed that paired populations in oil-polluted versus not-polluted habitats diverged independently in two different watersheds. Morphometrics revealed some divergence (particularly in head shape) between these environments, some of which was parallel between rivers. Reciprocal transplant experiments in nature, however, found little evidence of local adaptation based on survival and growth. Moreover, subsequent laboratory experiments showed that the two populations from oil-polluted sites showed only weak local adaptation even when compared to guppies from oil-free northern Trinidad. We conclude that guppies show little local adaptation to oil pollution, which might result from the challenges associated with adaptation to particularly stressful environments. It might also reflect genetic drift owing to small population sizes and/or high gene flow between environments.

## Introduction

Contemporary anthropogenic impacts, such as climate warming, habitat fragmentation, and pollution, can pose severe challenges for natural populations (Vitousek et al. 1997; Palumbi 2001; Stockwell et al. 2003). One challenge is that environmental change renders populations poorly adapted for the new environment, which can cause population declines and extirpations (Pimm et al. 1995; Hughes et al. 1997). Populations can arrest these declines by showing rapid (contemporary) adaptation to the new conditions, so-called evolutionary rescue (Gomulkiewicz and Holt 1995; Gonzalez and Bell 2013; Carlson et al. 2014). Meta-analyses have revealed that many populations show substantial adaptive evolution in response to anthropogenic disturbances (Hendry and Kinnison 1999; Reznick and Ghalambor 2001; Hendry et al. 2008), yet frequent extirpations also indicate that evolutionary rescue is not inevitable. Thus, a particularly important research goal for conservation and management is to understand how and why natural populations succeed or fail to adapt to environmental change.

The ability of a population to respond to environmental change will depend not only on its inherent adaptive potential but also on the severity of the disturbance. For instance, environmental impacts that are inherently ‘stressful’ (i.e., they cause a drastic imbalance between environmental demands and the response capabilities of the organisms) (Evans and Cohen 1987) can have complicating effects on adaptation and thus make adaptive responses particularly difficult (Hoffmann and Parsons 1997; Badyaev 2005; Gonzalez and Bell 2013). One reason is that fitness in stressful environments can be low even for well-adapted individuals, leading to small population sizes that increase drift and inbreeding (Frankham 1995; Willi et al. 2006), which can further decrease adaptation and fitness (Falk et al. 2012). As a result, the process of attempting to adapt to a stressful environment might lead to comparably low fitness of that population in all environments (Brady 2013). In short, adaptation to stressful environments might entail complex detrimental effects causing deviations from the typical expectations of local adaptation and leading to poor overall performance of affected populations. Of course, the existence of extremophile organisms shows that adaptation to very stressful environments is sometimes possible (e.g., Rothschild and Mancinelli 2001) and so adaptation to stressful environments can run the gamut from unqualified success to unqualified failure and everything in between.

An extreme case of environmental stress that might complicate local adaptation is severe chemical pollution. On the one hand, a number of studies have demonstrated local adaptation and the evolution of tolerance to pollutants such as heavy metals, pesticides, and soot (Macnair 1987; Taylor and Feyereisen 1996; Reznick and Ghalambor 2001; Ownby et al. 2002; Meyer and Di Giulio 2003; Williams and Oleksiak 2008). On the other hand, many species seem incapable of adapting to such conditions; that is, populations experiencing pollution are often very small and ecosystems under chronic exposure often suffer drastic reductions in species richness, community integrity, and productivity (Klerks and Weis 1987; Lotze and Milewski 2004; Revenga et al. 2005; Johnston and Roberts 2009). To gain insight into the process of adaptation (or the lack thereof) to chemically polluted environments, we here examine populations exposed to crude oil.

Crude oil can be a major contaminant in aquatic ecosystems. In particular, many crude-oil polycyclic aromatic hydrocarbons (PAHs) have mutagenic, carcinogenic, and toxic effects on organisms (Samanta et al. 2002; Hylland 2006). When considering the effects of PAHs in nature, the tendency has been to focus on major oil spills, such as the Exxon Valdez and Deep Water Horizon catastrophes (Peterson et al. 2003; Fodrie and Heck 2011). However, another important route to PAH pollution is smaller spills and local contamination events that occur during resource exploitation (e.g., oil sands: Schindler 2010; Kelly and Schindler 2010). Of particular utility for studying local (mal)adaptation, local contamination events usually affect only some populations of a species, whereas other nearby populations often remain unaffected. Local contamination events thus provide excellent opportunities to study the effects of stress on divergence and local adaptation through replicated reciprocal transplant experiments (Meyer and Di Giulio 2003; Tobler et al. 2008; Plath et al. 2013).

We here consider the possibility of local adaptation to crude-oil PAH pollution in natural populations of Trinidadian guppies (*Poecilia reticulata*)—a suitable system for several reasons. First, guppies show strong local adaptation to a broad range of environmental conditions, with examples including differential predation, canopy cover, and salinity (reviews: Endler 1995; Reznick and Ghalambor 2001; Magurran 2005). Second, adaptation to these environments often occurs independently in multiple watersheds, thus providing useful evolutionary replication and opportunities to test for parallel and convergent evolution (e.g., Reznick and Bryga 1996). Third, the extent of crude-oil pollution in guppy habitats varies dramatically on the scale of the entire island of Trinidad (absent in the north, but common in the south) and within specific rivers (for a given southern river, some tributaries can be heavily polluted whereas others are not). Fourth, guppies are one of the few fish species that can (often) tolerate extensive pollution (e.g., industrial effluents: Araujo et al. 2009), thus forming local populations in both oil-polluted and not-polluted habitats.

We started by identifying two rivers in southern Trinidad that contained abundant guppy populations in both oil-polluted and not-polluted tributaries. Differential pollution and bioavailability of contaminants were confirmed by gas chromatography (GC) and mass spectrometry of water samples and also via semipermeable membrane devices deployed at the sites. We then used neutral genetic markers to establish the likely evolutionary independence of oil-polluted versus not-polluted divergence in the two rivers. Next, a series of assays for adaptation were conducted on wild-caught fish. Although the use of wild-caught fish means that the genetic versus plastic contributions to divergence cannot be separated, this approach is the logical way to begin work in a new study system. Indeed, 14 of the 21 (66.7%) animal studies reviewed in a meta-analysis of local adaptation (Hereford 2009) used wild-caught individuals. Moreover, local adaptation at the genetic level (i.e., when using individuals from a common garden) is normally only anticipated when it is first revealed at the phenotypic level (i.e., when using individuals from the wild). For our phenotypic analyses, we first considered body shape differences between guppies from oil-polluted versus not-polluted habitats, as well as the extent to which any such differences were convergent/parallel between the rivers. Finding some evidence for convergence in body shape, we tested for local adaptation by reciprocally transplanting fish between oil-polluted and not-polluted habitats in the two rivers. Failing to find consistent evidence of local adaptation on this small scale, we next performed a laboratory-based reciprocal transplant experiment using guppies from southern (oil polluted) and northern (oil free) regions. Finding only modest evidence for local adaptation measured even on this larger scale, we venture a series of inferences regarding the effect of stressful environments on local adaptation.

## Methods

### Study sites

Our primary study sites were located in southern Trinidad, where crude oil is commercially exploited and pollutes rivers due to soil leakage and spillage (Sutton 1955; Agard et al. 1993).

**Figure 1:**
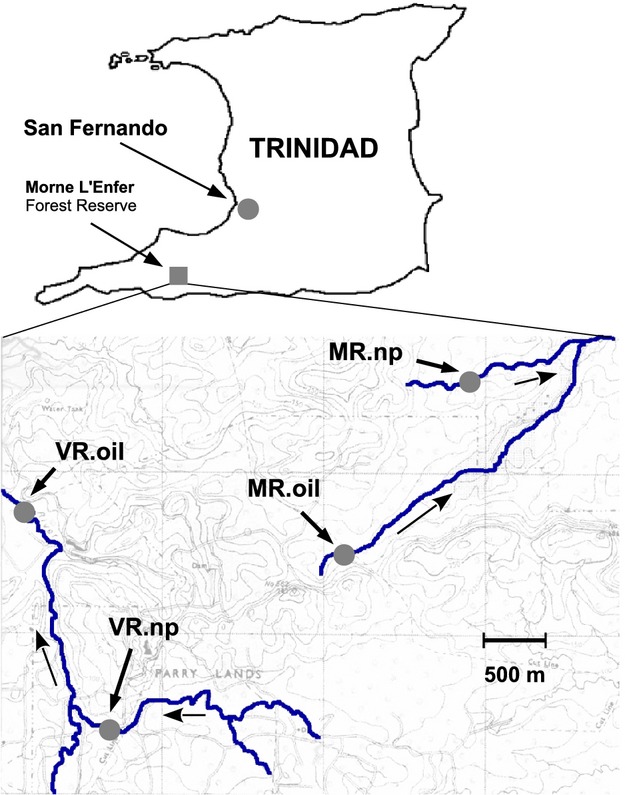
Map depicting rivers and field sites at Morne L' Enfer Forest Reserve, southern Trinidad: MR.oil (10°11′25.79 N/61°36′27.38°W); MR.np (10°11′40.58°N/61°36′12.65°W); VR.oil (10°11′23.84°N/61°37′34.54°W); and VR.np (10°10′26.78°N/61°37′31.95°W). Concave arrows indicate flow direction. See also Fig. S11 for pictures illustrating the extent of crude-oil pollution in the studied river systems due to above and below ground seepage.

In 2011 and 2012, we focused on guppies from two river systems, the Morne L' Enfer River (hereafter Morne River, MR) and the Vance River (VR), both running through the Morne L' Enfer Forest Reserve (Fig.1). These rivers were chosen because (i) they are remote from settled areas and thus were expected to lack anthropogenic contaminants other than crude oil, (ii) they include spatially separated oil-polluted and not-polluted tributaries, and (iii) they are separate drainages such that guppy divergence within each should be independent. The study sites contained few other fish species (only killifish, *Rivulus hartii*, and characins, *Astyanax* sp.) and were therefore considered to be low-predation environments (*sensu* Seghers 1973; Magurran 2005). Following the identification of suitable sites in each river, we sampled guppies from oil-polluted and not-polluted tributaries: Morne River, oil polluted (MR.oil; areal extent of pollution: ∼100 m up- and downstream from sampling site); Morne River, not polluted (MR.np); Vance River, oil polluted (VR.oil; areal extent of pollution: ∼50 m upstream and ∼10 0 m downstream from sampling site); and Vance River, not polluted (VR.np). The extent of pollution in both years and the bioavailability of contaminants for fish in these habitats were examined by (i) deploying semipermeable membrane devices (SPMDs) as passive samplers (Huckins et al. 1990) at each site in 2011, and (ii) conducting detailed gas chromatographic (GC) analyses of water samples taken in 2012 (details on petroleum hydrocarbon analyses and results are given in the supplementary material).

For some comparisons, we also sampled guppies from low-predation sites in the Aripo River (Ar) and Paria River (Pa) in the Northern Range mountains of Trinidad. The motivation in adding these two sites was to enable comparisons with populations certain to be from an oil-free region. By contrast, guppies from not-polluted sites from the southern streams could well have experienced oil pollution in their recent evolutionary history—or through recent gene flow with populations from oil-polluted habitats. Further rationale is provided below.

### Population genetics

Our goal in conducting genetic analysis was to examine population structure, gene flow, and evolutionary history. For this purpose, highly variable microsatellite markers were deemed appropriate. Future analyses of the genomic basis of adaptation to oil pollution would require other approaches.

Genomic DNA was extracted from fin clips collected in 2011 from the four southern sites (n_MR.oil_ = 38, n_MR.np_ = 21, n_VR.oil_ = 65, n_VR.np_ = 36) using the protocol of Elphinstone et al. (2003), with modifications to accommodate use of a Perkin Elmer MPII liquid-handling robot. All individuals were genotyped at 10 highly variable tetranucleotide microsatellite markers (Pre8, Pre9, Pre15, Pre26, Pre27, Pre28, Pre38, Pre80, g145, and g289; for details see Paterson et al. 2005). Within-population tests for linkage disequilibrium for all pairwise locus combinations and for deviations from Hardy–Weinberg equilibrium (HWE) were performed using the software GENEPOP (Raymond and Rousset 1995; Rousset 2008), with all p-values corrected for multiple comparisons based on the false discovery rate (Benjamini and Hochberg 1995). None of the pairs of loci showed significant linkage disequilibrium (all *P* > 0.4, exact test for genotypic disequilibrium), and summary statistics for all 10 loci are given in Table S1. Two loci (Pre27 and Pre28) showed significant deviations from HWE in two populations (Table S1), and we therefore performed all analyses twice: once for the full set of 10 loci and once for the subset of eight loci confirmed to be in HWE (excluding Pre27 and Pre28). Results did not change qualitatively between the two analyses, and we therefore only report the results based on the full set of 10 loci.

Genetic variation and population structure among the four southern populations were examined in several analyses. First, *F*_ST_ (Weir and Cockerham 1984) and Jost' s D (Jost 2008) were calculated between all population pairs and tested for significance using permutations (*n* = 1000) of genotypes across individuals. Second, discriminant analysis of principal components (DAPCs) was implemented using the R package adegenet (Jombart 2008; R Development Core Team 2009; Jombart et al. 2010). DAPC first centers and scales genetic data and performs a principal component analysis from which the axes of maximal variance are extracted. These variables are then subjected to linear discriminant analysis allowing the representation of populations in genotypic space. DAPC is robust against HWE deviations and makes no assumptions regarding the underlying data structure or population genetic model (Jombart et al. 2010). Third, we conducted a Bayesian admixture model analysis using STRUCTURE 2.3 (Pritchard et al. 2000) with sample sites as informative priors (LOCPRIOR, Hubisz et al. 2009). STRUCTURE was run for 10 separate MCMC simulations over 50 000 burn-ins with 100 000 repeats for each *k*, including *k* = 1 to *k* = 6 (the assumed number of populations plus two). The most likely number of k clusters was estimated based on the Δk criterion (Evanno et al. 2005), and the respective MCMC runs were merged using the software CLUMPP (Jakobsson and Rosenberg 2007).

Gene flow between oil-polluted and not-polluted habitats within each river system was assessed as the ratio of immigration rate to mutation rate (M = m/μ). We used a Bayesian framework in the software package MIGRATE (Beerli and Felsenstein 2001; Beerli and Palczewski 2010), which estimates the mutation-scaled population size parameter Θ and the mutation rate parameter M, which can then be used together with the mutation rate (μ) to calculate effective population size (N_e_) via Θ = 4 N_e_ μ, as well as the number of new variants introduced by immigration relative to mutation (m) via M = m/μ. For our calculations of N_e_, we used a mutation rate of μ = 5.56 × 10^−4^, a common value for microsatellites (Whittaker et al. 2003; Yue et al. 2007). We applied a Brownian motion approximation to the stepwise mutation model for microsatellite data and performed a Bayesian inference search strategy using a constant mutation rate and an exponentially distributed prior. A slice-sampling MCMC algorithm was used with a burn-in of 100 000 iterations and 10 000 recorded steps. We tested the migration models with a Θ value estimated from *F*_ST_ calculations, and migration between oil-polluted and not-polluted sites in the respective rivers was free to vary across all loci. An additional analysis of migration rates within each river was conducted based on an isolation-with-migration model implemented in IMa2 (Hey and Nielsen 2007) applying a geometric heating scheme for 10^6^ steps after a burn-in period of 10^6^. Five independent IMa2 runs starting with varying random seeds produced similar posterior distributions.

### Morphometrics

From each of the four southern sites, adult males collected in 2011 (n_MR.oil_ = 53, n_MR.np_ = 48, n_VR.oil_ = 26, n_VR.np_ = 38) were used for morphometric analyses. Males were used rather than females so as to avoid effects of gravidity on body shape, as is common in analyses of live-bearing fishes. Landmark data were collected from digital photographs (Fig. S1) using the imaging software CLIC/coo (Dujardin et al. 2010). The landmark data were then divided into (i) whole fish shape (LM set 1), and (ii) head morphology (LM set 2, Fig. S1). The analysis of head shape was added because preliminary field observations suggested the possibility of interpopulation differences in head shape and because gill (and head) shape varies among fish populations in response to variation in water quality (e.g., Laurent and Perry 1991; Tobler et al. 2011). For comparative purposes, the same morphometric variables were obtained for 32 male guppies collected from the Paria River (Pa) in 2012.

Shape variables were extracted from two-dimensional shape coordinates of landmarks using generalized Procrustes analysis (GPA). GPA scales landmark configurations to centroid size, which is the square root of the sum of squared distances of landmarks to their centroid (Bookstein 1991). After rotation of the scaled landmark sets in reference to the calculated mean shape, residual differences were subjected to principal component analysis (PCA) to obtain axes that depict maximal variation (i.e., ‘morphospace’, Adams et al. 2004). GPA was performed in R applying the algorithms described in Claude (2008). The resulting principal components were used in a MANCOVA model, including centroid size as a covariate, to test for shape differences among the five populations. MANCOVAs were tested for significance using the Pillai–Bartlett statistic (Hand and Taylor 1987). To scrutinize the degree of parallelism in shape differences between oil-polluted and not-polluted sites in the two southern rivers (MR.oil vs. MR.np, and VR.oil vs. VR.np), we performed a canonical variate analysis (CVA) on the dependent variables in the MANCOVA model to compute a linear transformation of variables into canonical space representing maximal separation among groups (Scheiner 2001). Scores on the first CV axis were then used to depict any parallel trends in morphology (e.g., Langerhans and DeWitt 2004).

### Classification-based analysis of exchangeability

We considered how well guppies from the four southern populations could be assigned to each population based on their phenotypes and genotypes: that is, their ‘exchangeability’ *sensu* Hendry et al. (2013). This analysis used the individual posterior classifications calculated from three DAPC analyses: two for morphological traits (full body shape or head shape) and one for the 10 neutral microsatellites. For all DAPCs, we used a score analysis (Jombart et al. 2010) to determine the optimal number of retained principal components: 10 for full body shape, five for head shape, and 16 for neutral markers. We then categorized individual classification scores into the ‘candidate populations’ in four possible categories (see Hendry et al. 2013 for details on this approach): the population of origin (Origin), a parapatric population in a different habitat (an individual from an oil-polluted site to the population from a not-polluted site, or vice versa, within the same river), an allopatric population in a similar habitat (oil polluted to oil polluted or not polluted to not polluted, between rivers), and an allopatric population in a different habitat (oil polluted to not polluted or not polluted to oil polluted, between rivers). High classification scores to an allopatric population in a similar habitat would suggest parallel responses to similar selection regimes in the two rivers, whereas high classification scores to the parapatric population in the different habitat would imply an important role of gene flow between habitats within a river (Hendry et al. 2013).

### Reciprocal transplants in the field

Field experiments in 2011 used guppies from the four southern populations (Fig.1): Morne River, not polluted (MR.np); Vance River, not polluted (VR.np); Morne River, oil polluted (MR.oil); and Vance River, oil polluted (VR.oil). From each of these sites, we collected 80–100 similarly sized adult females to be transplanted into field enclosures. Females were used because we wanted to use growth as one fitness surrogate and males stop growing after maturity. Juveniles were not used as we were concerned they might be too small and fragile for the experimental procedures. Immediately after capture, all experimental fish were brought to the laboratory where they were anaesthetized with MS 222 to weigh them and to mark them individually using elastomer dyes (Reznick et al. 1996; Weese et al. 2010). During this 2–3 day marking period, the fish were kept in aerated tanks with ad libitum food.

In each of the four field sites, we placed five cylindrical wire-mesh enclosures (height = 50 cm, diameter = 35 cm, mesh size = ∼2 mm) per test population, for a total of 20 enclosures per site and 80 enclosures across all sites. The enclosures were placed so that they floated at the surface, with approximately 80% of the total enclosure volume submerged. This particular design was chosen because guppies in these sites are frequently observed in the top/middle of the water column, which is also where our SPMDs were placed. (It would also be interesting in future work to allow guppies access to the soil–water interface.) Each enclosure was stocked with four or five (depending on availability) of the above-described females. The enclosures were monitored every second day for mortality, and dead fish were replaced with newly caught females so as to maintain constant densities. On the sixth day (a suitable time period for jointly estimating mortality and growth), all of the fish were removed, identified by inspection of their marks, and weighed.

Fitness surrogates (survival and mass change) were analyzed with (generalized) linear mixed effect models (G)LMMs in the *lme4* package in R (R Development Core Team 2009; Bates et al. 2014). The survival analyses used a GLMM with a binomial link function and included a total of 416 fish (n_MR.oil_ = 88, n_MR.np_ = 109, n_VR.oil_ = 88, n_VR.np_ = 131). The mass change analyses used a LMM that included the 261 survivors (n_MR.oil_ = 39, n_MR.np_ = 61, n_VR.oil_ = 81, n_VR.np_ = 80). All (G)LMMs incorporated initial mass as a covariate, a random effect structure accounting for experimental units (individual enclosures), and fixed effect predictors corresponding to specific hypotheses (see below). Statistical significance of the predictor coefficients was estimated based on ANOVA tables, and likelihood-ratio tests (Zuur et al. 2009) and predictor effects were investigated through fitted values under the model. In particular, we constructed two separate (G)LMMs for each fitness surrogate so as to investigate (i) ‘environment-level adaptation’, and (ii) ‘population-level local adaptation’, as well as other hypotheses (see below). All of the tested hypotheses are illustrated in Fig. S2.

Environment-level adaptation (hypothesis 1) corresponds to the expectation that fitness will be higher in transfers of fish between similar environments than in transfers of fish between different environments: that is, a test for adaptation to oil pollution independent of the specific river. The fixed effects of interest were the ‘Oil test site’ predictor, whether or not oil pollution was present at the test site (i.e., overall effects of oil pollution); the ‘Oil origin’ predictor, whether or not oil pollution was present at the home-site of the population (i.e., effect of oil pollution in the environment of origin); and the ‘Oil test site x Oil origin’ interaction term (i.e., general adaptation to oil pollution). The random effect structure accounted for population origin and enclosures nested within sites.

Population-level local adaptation (hypothesis 2) corresponds to the expectation that fitness will be higher for native individuals at their home-site than for non-native individuals at the same site (Endler 1986; Schluter 2000; Kawecki and Ebert 2004). The fixed effects of interest were the ‘Population’ predictor, specifying the population origin; the ‘Site’ predictor, specifying the test site; and the ‘Population × Site’ interaction term (i.e. local adaptation). The random effect structure accounted for enclosures nested within sites. Moreover, we tested for ‘population-specific superiority’ (hypothesis 3) that invokes the criteria above but asks whether fitness effects are substantiated for each of the four populations individually. That is, some populations might show home-site superiority whereas others might not. This hypothesis was addressed using separate (G)LMMs in which the contrast for each population' s performance in its native site versus the remaining three sites was represented as fixed effect predictor, and the random effect structure accounted for enclosures nested within sites. Finally, the expectation that local adaptation to one environment comes at the cost of adaptation to other environments (i.e., fitness trade-off) was considered by comparing fitness in the native habitat versus fitness in the non-native habitat (see Hereford 2009).

### Reciprocal transplants in the laboratory

The above field experiment did not reveal strong differences among populations (see Results), which might occur because the entire southern region has a long history of oil pollution that varies in space and time. Therefore, the next logical step was to design a new transplant experiment that also included populations from a region in Trinidad that has no history of oil pollution: the Northern Range mountains. The 2012 experiment therefore used fish from the two polluted sites in the 2011 experiment (MR.oil and VR.oil) and from two northern populations: the Paria River (Pa) and the Aripo River (Ar). As in the 2011 experiments, all fish were individually marked with elastomer dyes and monitored for 2–3 days before the experiment. For the experiment itself, we used eight glass aquaria (length = 150 cm, height = 50 cm, width = 35 cm) representing two replicate mesocosms (blocks) for each population' s environment. Specifically, aquaria that were filled with water, rocks, and sediment were transported from the four field sites (Mr.oil, VR.oil, Pa, and Ar) to the laboratory at The University of the West Indies, Trinidad (UWI). Every second day, we exchanged ∼ one-fourth of the water in each tank with newly collected water from the respective field sites. To start the experiment, individual aquaria were stocked with six or seven (depending on availability) experimental fish from each population (24–28 fish per aquarium) in a reciprocal design. Aquaria were monitored for mortality three times a day over a period of 6 days. Dead fish were replaced by new experimental fish to maintain densities and to restore sample sizes in cases of early death (i.e., within the first 12 h of exposure). Replacement fish were treated in the same way as described above. In total, we collected survival data on 409 fish (n_MR.oil_ = 103, n_VR.oil_ = 103, n_Pa_ = 99, n_Ar_ = 104) and mass change data on the 231 surviving fish (n_MR.oil_ = 36, n_VR.oil_ = 60, n_Pa_ = 46, n_Ar_ = 89).

Fitness surrogates (survival and mass change) for the laboratory experiments were analyzed using LMMs following the same logic as explained above for the field experiments (see hypotheses 1–3 above). Additionally, the laboratory experiment generated detailed time-series data, yielding the number of hours survived per fish. Results did not differ between the two survival metrics (dichotomous survived/died vs. time-series hours survived), and so we only report results from the more comprehensive latter time-series models. Random effect structures for ‘environment-level adaptation’ (hypothesis 1) accounted for population origin and aquaria nested within blocks. Random effect structures for ‘population-level local adaptation’ (hypothesis 2) and ‘population-specific superiority’ (hypothesis 3) accounted for aquaria nested within blocks. All LMMs incorporated initial mass as a covariate. Moreover, we used stratified Cox proportional hazard (CPH) models that incorporated the population-within-site structure as strata. Here, significance was inferred by testing every pairwise population contrast in each testing environment separately. In addition to CPH analyses with all four study populations (VR.oil, MR.oil, Paria, and Aripo), we tested for regional differences in separate CPH models for Northern Range fish (Aripo and Paria together) and southern fish (MR.oil VR.oil together).

### Comparison to meta-analysis

We used growth and survival from the reciprocal transplant experiments (field and laboratory) to quantitate local adaptation following the logic of Hereford (2009). In particular, local adaptation of a given population at its homesite was quantified as LA = (W_native population_ − W_non-native population_) / avg (W_site_), where W represents the mean fitness of a population at a given site and avg (W) represents the mean fitness of all populations at that site. We compared these LA estimates from our study populations to the distribution of LA estimates from a recent survey of published studies of local adaptation (Hereford 2009).

## Results

### Contamination

Chemical analysis of the SPMDs deployed in 2011 revealed a wide range of PAH compounds that are bioavailable to fish at the two oil-polluted sites (MR.oil and VR.oil) but not at the two not-polluted sites (MR.np and VR.np). Chemicals detected included, among others, branched alkylbenzenes and alkylnaphthalenes, alkylphenanthrenes, and dibenzothiophenes (see supplemental material for details). These compound classes are known to be toxic, mutagenic, and carcinogenic and to have detrimental effects on the development in fish and other vertebrates (Moles and Rice 1983; Serigstad 1987; Heintz et al. 1999; Samanta et al. 2002; Neff 2003; Hylland 2006; Johnson et al. 2008). Supporting these findings, GC analyses focusing on the total petroleum hydrocarbon (TPH) concentration of water samples taken at the field sites in 2012 indicated high levels of hydrocarbon contamination at MR.oil (57.58 ± 8.09 mg/L) and VR.oil (214.8 ± 37.87 mg/L). Importantly, these levels are well within the range of hydrocarbon contamination previously reported to be harmful to a variety of fish species (NCM 2007; Hook et al. 2010; Milinkovitch et al. 2013; Crowe et al. 2014). Together, these results make clear that harmful crude-oil contaminants are present at these sites and are bioavailable to local fish.

### Population genetics

**Figure 2:**
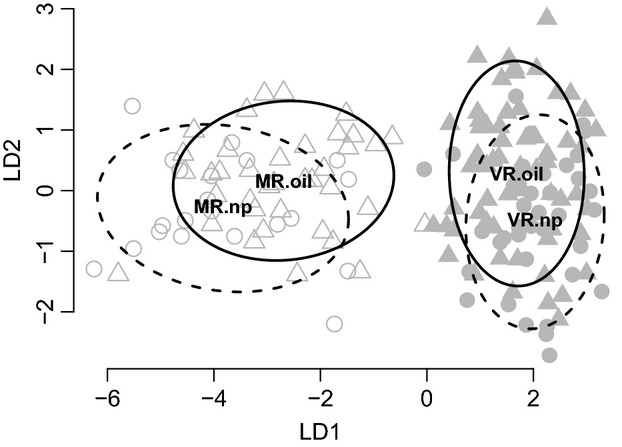
Linear discriminant analysis of principal components (DAPCs) summarizing genetic variation among the four studied populations from southern Trinidad (MR.oil, MR.np, VR.oil, and VR.np). Ellipses depict 75% data envelopes (solid lines: Morne River, dashed lines: Vance River), and bold letters indicate the respective population centroids.

A variety of analyses all indicate that guppy populations in the Morne River (MR) are genetically distinct from those in the Vance River (VR), consistent with expectations of independent origins of population divergence within each. Discriminant analysis of principal components revealed a strong genetic separation between the two rivers, with MR populations clustered separately from VR populations along the LD1 axis (Fig.2). Additional minor differentiation along the LD2 axis was evident between oil-polluted and not-polluted populations in VR but not in MR (Fig.2). These patterns were supported by Bayesian cluster analyses in STRUCTURE, where the most likely number of populations was *k* = 2 (Fig. S3), reflecting the clear separation between VR and MR (Fig. S4A). Running STRUCTURE for *k* = 4, further confirmed notable genetic differentiation between rivers but no genetic differentiation with them. (Fig. S4B). Pairwise *F*_ST_ and Jost' s D values corroborated these results in showing a strong separation between rivers, with no divergence between environments within rivers (Table1).

**Table 1:** Pairwise *F*_ST_ (lower diagonal) and Jost' s D (upper diagonal) comparisons between the four studied populations from the southern region

Site	MR.oil	MR.np	VR.oil	VR.np
MR.oil	–	0.028	0.351*	0.395*
MR.np	0.004	–	0.489*	0.521*
VR.oil	0.051*	0.078*	–	0.015
VR.np	0.056*	0.081*	0.002	–

*P*-values (^*^indicates *P* < 0.001) were obtained through permutation (*n* = 1000) of genotypes among individuals.

As estimated in MIGRATE, gene flow between oil-polluted and not-polluted sites was higher in VR than in MR and was higher from oil-polluted into not-polluted sites than in the reverse direction in both rivers (m_into_ _VR.oil_ = 0.010 vs. m_into VR.np_ = 0.390; m_into MR.oil_ = 0.026 vs. m_into MR.np_ = 0.047). These patterns were confirmed through analyses in IMa2 (Fig. S5). Effective population sizes (Ne) did not differ among the populations (Ne_MR.oil_ = 44.3, Ne_MR.np_ = 43.5, Ne_VR.oil_ = 44.0, Ne_VR.np_ = 44.2). Inbreeding coefficients were higher in MR than VR but did not differ between oil-polluted and not-polluted populations within rivers (Fis_MR.oil_ = 0.015, Fis_MR.np_ = 0.014, Fis_VR.oil_ = 0.050, Fis_VR.np_ = 0.056). Expected heterozygosity (He) was similar among all populations (0.834 < He < 0.878) and comparable to He found in other guppy populations (e.g., Kelly et al. 1999).

### Morphometrics

**Figure 3:**
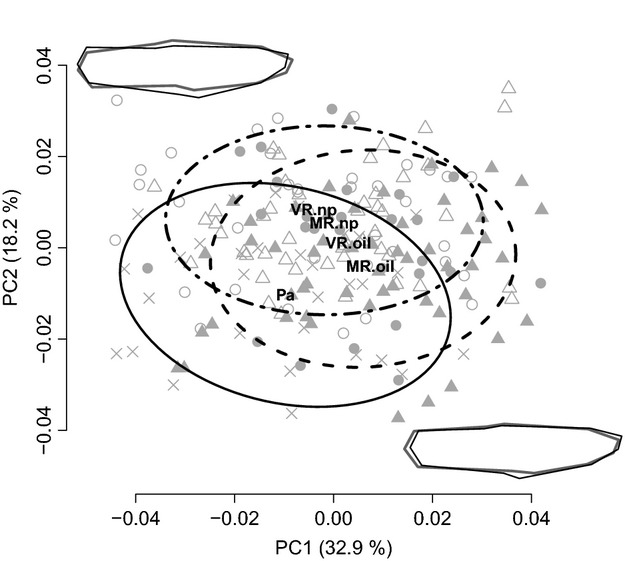
Full body shape principal component scores (PC1–2) for guppies from the four southern populations (MR.oil, MR.np, VR.oil, and VR.np) and the Paria River (Pa) from northern Trinidad. Body shape outlines next to the axes indicate shape change along the particular PC with thin black lines depicting the maximum and bold gray lines depicting the minimum for each component. Ellipses are 75% data envelopes covering the Paria population (cross-symbols and solid line), both populations from oil-polluted habitats (MR.oil and VR.oil; filled triangles, filled circles, and dashed line), and both populations from not-polluted habitats (MR.np and VR.np; open triangles, open circles, and dash-dotted line). Bold letters indicate the respective population centroids.

MANCOVA based on the first 15 axes from the Procrustes PCA on body shape—accounting for 98.9% of the total variance—revealed significant differences among the five populations (Pillai = 1.32, df = 4, *F* = 5.91, *P* ≪ 0.001). The most striking pattern was a separation of guppies in the Paria River in northern Trinidad from those in the four southern populations. This separation was confirmed through separate ANCOVAs on the first two PC axes (PC1: *F* = 4.85, df = 4, *P* < 0.001; PC2: *F* = 10.87, df = 4, *P* < 0.001; Fig.3). In particular, Paria males had more positive values for PC1, corresponding to a shorter and less streamlined body shape. Paria males also had more negative values for PC2, corresponding to shallower bodies (Fig.3). Focusing on southern populations only, Procrustes PCA showed a weak parallel pattern of divergence in morphospace, such that within-river divergence was in a similar direction between oil-polluted and not-polluted populations (Fig.3). This trend toward parallelism was corroborated in the CVA of the first 15 PC axes (Fig. S6A). Specifically, guppies from oil-polluted sites have slightly longer and shallower (more streamlined) bodies than do guppies from not-polluted sites (VR.np and MR.np).

**Figure 4:**
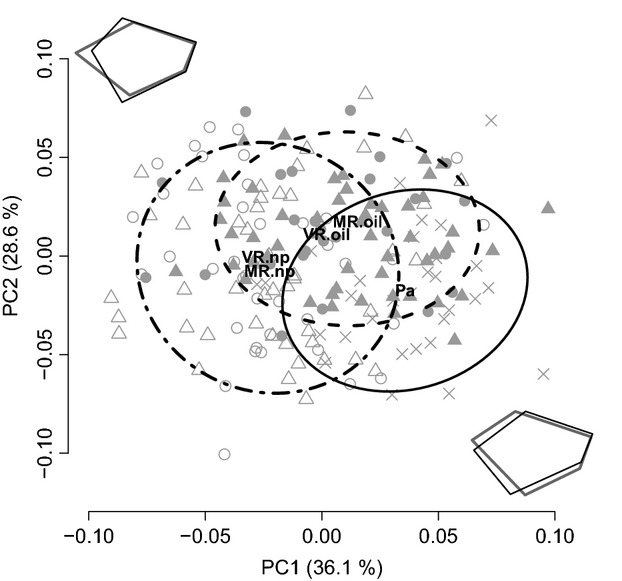
Head shape principal component scores (PC1–2) for guppies from the four southern populations (MR.oil, MR.np, VR.oil, and VR.np) and the Paria River (Pa) from northern Trinidad. Head shape outlines next to the axes indicate shape change along the particular PC with thin black lines depicting the maximum and bold gray lines depicting the minimum for each component. Ellipses are 75% data envelopes covering the Paria population (cross-symbols and solid line), both populations from oil-polluted habitats (MR.oil and VR.oil; filled triangles, filled circles, and dashed line), and both populations from not-polluted habitats (MR.np and VR.np; open triangles, open circles, and dash-dotted line). Bold letters indicate the respective population centroids.

The above weak trend toward parallelism between guppies from oil-polluted versus not-polluted sites became stronger when analyses were restricted to the subset of landmarks that described head morphology (Fig S1). In this case, MANCOVA including the first five PC axes—accounting for 97.1% of the variance—again revealed significant differences among the populations (Pillai = 0.72, df = 4, *F* = 8.28, P ≪ 0.001). Moreover, the two oil-polluted populations clustered together and somewhat separately from the two not-polluted populations, a trend that was confirmed when the first two PCs were analyzed separately in ANCOVAs (PC1: *F* = 22.12, df = 4, *P* < 0.001; PC2: *F* = 6.22, df = 4, *P* < 0.001; Fig.4). Interpretation of shape variation on the first two PCs, as well as CVA of the first five PC axes, indicated that guppies from oil-polluted sites had deeper heads compared to guppies from not-polluted sites (Fig.4, Fig. S6B).

### Classification-based analysis of exchangeability

**Figure 5:**
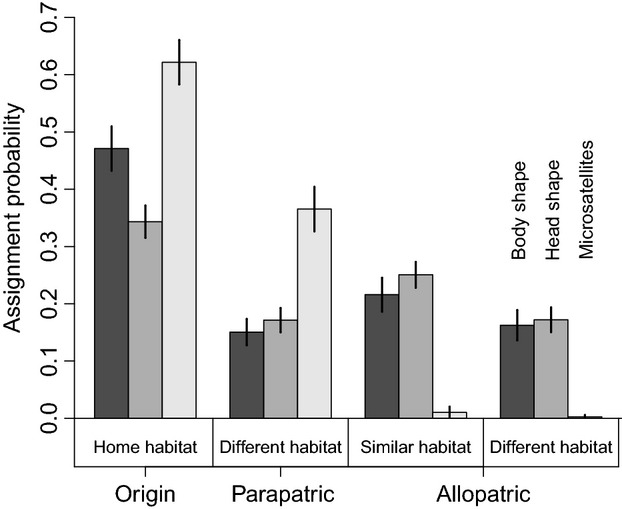
Average classification success based on individual assignment scores from separate discriminant analyses of principal components (DAPCs) for three variable classes (body shape, head shape, and microsatellites). Labels for the one set of bars apply in the same order to each set of bars and values depict means with 95% CIs.

Analysis of southern populations based on DAPCs revealed that classification probabilities were highest to the population of origin for all variable classes: body shape, head shape, and microsatellites (Fig.5). This pattern indicates that all of the populations were, to at least some extent, differentiated for these variable classes (all *P* < 0.005, pairwise t test of classification probabilities with false discovery rate p-value adjustment). For genetic markers, exchangeability (i.e., classification to a nonorigin population) was highest to the parapatric population of the other habitat type in the same river (all *P* < 0.010), which likely reflects shared origins and ongoing gene flow within but not between rivers. For morphology, exchangeability was highest between populations in similar habitats in different rivers (all *P* < 0.010). This last result suggests that selection regimes and growth conditions acting between oil-polluted versus not-polluted environments are to some extent parallel across rivers.

### Reciprocal transplants in the field

**Figure 6:**
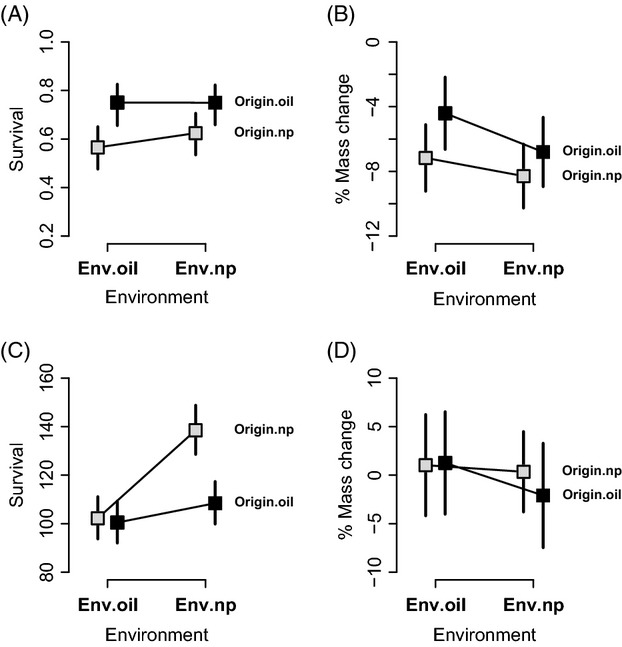
Environment-level adaptation in reciprocal transplant experiments: 2011 in the field (upper panel) and 2012 in the laboratory (lower panel). Figures depict average population contrasts (+95% CI) between oil-polluted and not-polluted environments estimated from (G)LMMs for survival (A and C) and mass change (B and D) as fitness surrogates (see Methods for details). Gray rectangles depict populations from not-polluted (Origin.np) environments and black rectangles depict populations from oil-polluted (Origin.oil) environments.

Reciprocal transplants in the field revealed high mortality within 6 days for three populations (MR.oil = 55.7%, MR.np = 44.0%, VR.np = 39%) but low mortality for the fourth (VR.oil = 8%). Strong evidence for adaptation to oil pollution was absent for all three hypotheses tested. Hypothesis 1: no interaction was found between the ‘Oil test site’ predictor and the ‘Oil origin’ predictor for survival or mass change: that is, no environment-level adaptation to oil (Fig.6, Table2). Hypothesis 2: survival differences between populations were fairly consistent across test sites (e.g., VR.oil was always highest), such that no evidence was found for population-level local adaptation (high overall survival in specific home environments) tested by the interaction between the ‘Population’ predictor and the ‘Site’ predictor (Fig. S7, Table S2). Changes in mass were less consistent among populations across sites (Fig. S7) but also showed no sign of population-level local adaptation (Table S2). Hypothesis 3: population-specific tests for homesite superiority also failed to detect any local adaptation in survival or mass change (Table S3).

**Table 2:** Fixed effect predictors of environment-level adaptation (hypothesis 1, see Methods) in the 2011 field experiments. Values depict ANOVA summary statis tics of separate (G)LMMs for fitness surrogates survival and mass change. All models included initial mass as a covariate and a random effect structure that accounted for population origin and enclosures nested within sites

Predictor	Model	Sum Sq	*F*	χ²	*P*(χ²)
Oil origin	Survival	0.545	0.545	0.541	0.461
	Mass change	85.363	1.319	1.283	0.257
Oil test site	Survival	0.113	0.113	0.114	0.735
	Mass change	9.534	0.146	0.138	0.710
Interaction	Survival	0.292	0.292	0.289	0.591
	Mass change	25.767	0.405	0.405	0.524
Initial mass	Survival	0.135	0.134	0.128	0.721
	Mass change	234.867	3.766	3.767	0.052

### Reciprocal transplants in the laboratory

Reciprocal transplant experiments in the laboratory revealed high mortality for three populations (VR.oil = 41%, MR.oil = 65%, Pa = 53%) but lower for the fourth (Ar = 14%). Strong evidence for adaptation to oil pollution was absent (or at least limited) for all three hypotheses tested. Hypothesis 1: the interaction between the ‘Oil test site’ predictor and the ‘Oil origin’ predictor revealed a significant effect on survival but not mass change: that is, some environment-level adaptation but only for survival and notably mainly driven by higher survival in not-polluted (np) environments (Fig.6, Table3). In these same models, guppies in oil-polluted water as the test environment showed reduced survival but no significant mass change (tested by the ‘Oil test site’ predictor), and survival but not mass change was lower for guppies from oil-polluted sites (tested by the ‘Oil origin’ predictor) (Table3). Hypothesis 2: although mixed effect model analyses revealed an effect on survival and mass change for the interaction between the ‘Population’ predictor and the ‘Site’ predictor (Table S4), its significance was mainly driven by the high overall performance of the Ar, Pa, and VR.oil populations (no trade-off) and by the poor overall performance of the MR.oil population (Fig. S8). Hypothesis 3: the latter effect was confirmed by the lack of population-specific homesite superiority in performance of these three populations (VR.oil, Pa, and Ar) (Table S5). We therefore reject the hypothesis of population-level local adaptation to oil pollution.

**Table 3:** Fixed effect predictors of environment-level adaptation (hypothesis 1, see Methods) in the 2012 laboratory experiments. Values depict ANOVA summary statistics of separate LMMs for fitness surrogates survival and mass change. All models included initial mass as a covariate and a random effect structure that accounted for population origin and aquaria nested within blocks

Predictor	Model	Sum Sq	*F*	χ²	*P*(χ²)
Oil origin	Survival	2.885	0.719	0.742	0.389
	Mass change	36.860	0.035	0.042	0.838
Oil test site	Survival	50.960	11.447	11.218	8.1 × 10^−4^
	Mass change	84.640	0.321	0.262	0.609
Interaction	Survival	40.089	9.279	9.279	0.002
	Mass change	69.840	0.291	0.291	0.589
Initial mass	Survival	22.341	3.149	3.148	0.076
	Mass change	2147.350	9.221	9.221	0.002

Survival time analyses based on CPH models supported the above mixed effect model analyses. Pooling fish across regions (i.e., all fish) revealed higher mortality rates in oil-polluted aquaria than in not-polluted aquaria (*z* = 2.31, *P* = 0.021, Fig. S9). Pooling fish within regions (north vs. south) revealed that (i) fish from the not-polluted northern region (Ar and Pa) had higher mortality rates in oil-polluted water than in not-polluted water (*z* = 4.33, *P* < 0.001, Fig. S9), (ii) fish from the oil-polluted southern region (VR.oil and MR.oil) performed poorly in both water conditions, and (iii) southern fish showed no differences in mortality between oil-polluted and not-polluted aquaria (*z* = −1.16, *P* = 0.250, Fig. S9). Analyzing populations separately did not reveal strong evidence for local adaptation in the two populations from the southern region (VR.oil and MR.oil). Fish from the northern Aripo populations (Ar) survived longer than did those from the other populations in all four water conditions (Fig. S9). Fish from the VR.oil habitat performed better in their homesite condition than the other three populations, but this was not significantly different from the Ar fish, and VR.oil fish survived poorly in the other three conditions (Fig. S9). In the not-polluted aquaria, both populations from the southern region showed significantly higher mortality rates than did populations from the northern region (Fig. S9).

### Local adaptation metrics

**Figure 7:**
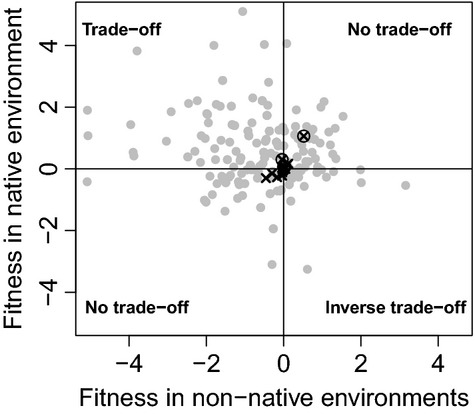
Plot of fitness advantages in native versus non-native sites for each population (•: data from a meta-analysis (Hereford 2009) conducted on 74 published studies; x: 2011 and 2012 reciprocal transplants; encircled crosses depict the VR.oil population). Fitness is depicted as pairwise difference relative to the mean fitness at each site. Quadrants represent alternative outcomes concerning fitness trade-offs. For details, see main text.

In the field experiment, the only instance of apparent local adaptation (LA) was the survival of VR.oil guppies (LA_survival_ = 1.06), which nevertheless did not show evidence of a fitness trade-off between environments (Fig.7). None of the other three populations showed positive LA, which instead ranged from −0.29 to −0.02 (mean_LA_ = −0.11). In the laboratory experiments, VR.oil showed positive LA (LA_mass change_ = 0.06, LA_survival_ = 0.33) and a fitness trade-off between environments (Fig.7), whereas MR.oil showed local maladaptation (LA_mass change_ = −0.05, LA_survival_ = −0.15). Aripo and Paria guppies showed positive local adaptation in survival (LA_survival, Aripo_ = 0.17, LA_survival, Paria_ = 0.06) but not in mass change (LA_mass change, Aripo_ = 0.00, LA_mass change, Paria_ = −0.11, Fig.7). Thus, the overall extent of local adaptation in our study was considerably lower than that reported in the meta-analysis of Hereford (2009) (Fig. S10).

## Discussion

Our key results can be summarized as follows. First, genetic differentiation at neutral markers was higher between watersheds than within them, suggesting independent origins of any oil-polluted versus not-polluted divergence. Second, guppies from oil-polluted versus not-polluted habitats showed some parallel divergence in body shape (especially head shape), suggesting some repeatable—that is ‘parallel’ or ‘convergent’ – responses. Third, reciprocal transplants (i) revealed detrimental effects of oil pollution (e.g., lower survival in oil-polluted habitats), but (ii) did not provide much consistent evidence of local adaptation to that pollution. These results stand in stark contrast to previous research on guppies showing extensive, repeatable, and rapidly evolving adaptive divergence between other types of environments (e.g., high predation versus low predation; high productivity versus low productivity) (Reznick and Bryga 1996; Reznick et al. 1997; Grether et al. 2001; O' Steen et al. 2002; Arendt and Reznick 2005; Gordon et al. 2009). While also considering other possibilities, we suggest that the apparent weakness of adaptation to oil pollution (especially in MR.oil guppies) might reflect the inherent difficulty of adapting to physiologically stressful abiotic conditions (Johnston and Roberts 2009; Bijlsma and Loeschcke 2012; Brady 2013).

### Whither adaptation?

Studies finding morphological differences between populations in different habitats typically infer that divergent selection has caused local adaptation—and this inference is particularly robust when the differences are parallel (or convergent) across multiple independent population pairs (Schluter 2000; Langerhans and DeWitt 2004). Our results fit this paradigm in that oil-polluted and not-polluted guppy populations showed morphological differences that are at least partially parallel between watersheds (Figs5, Fig S6): guppies from oil-polluted sites have shallower bodies and (especially) deeper and larger heads than do guppies from not-polluted sites (Fig.4). Differences in morphology, including head shape, in response to pollution have been reported for various fish species (Lindesjöö and Thulin 1992; Sun et al. 2009), where they might be related to hyperplasia, gill enlargement, and an excess of mucous cells in the gill region (Haaparanta et al. 1997; Fracácio and Verani 2003; Tkatcheva et al. 2004; Borg and Trombetta 2010). In particular, enlargement of the gill area and thickening of gill epithelium cells can increase the water–blood barrier and thus slow the uptake of pollutants (Fracácio and Verani 2003; Tkatcheva et al. 2004). Despite these tempting associations, it is not clear whether our results for head morphology are directly linked to changes in gill morphology, nor whether they constitute adaptive versus pathological responses, such as stunted growth due to poor living conditions.

In contrast to the above divergence in morphology, our reciprocal transplant experiments—in both the field and the laboratory—found little evidence for local adaptation; and what evidence we did find was weak and only evident at a large spatial scale. That is, even though oil contamination had negative effects on survival and mass change, these effects were not generally weaker for guppies from oil-polluted environments than for guppies from not-polluted environments (Fig.6, Fig. S7, Fig. S8). Indeed, quantitation of standard metrics of local adaptation (Hereford 2009) indicate that most populations were seemingly maladapted for their local environments (mean_LA_ = −0.11, Fig. S10). The only exception to this generalization was the VR.oil population, which showed some local adaptation with respect to survival (Fig. S7–S9). Overall, then, our results reinforce the key point (Hendry and Gonzalez 2008) that adaptation with respect to traits (e.g., head shape) is not the same as adaptation with respect to fitness components (e.g., survival and mass change). Stated another way, populations can show adaptive divergence in traits that will not necessarily have dramatic fitness consequences. The important question to consider next is why local adaptation in fitness components is absent or weak: we first consider methodological issues before turning to biological possibilities.

One concern is whether or not our experimental methodology was well suited to detect local adaptation—and we think it was for two main reasons. First, the experiments were certainly effective in detecting differences among populations (VR.oil showed the highest survival in multiple environments) and environments (oil-polluted environments reduced survival and growth). Second, we did not find strong evidence of local adaptation in either the laboratory or the field, or when using populations sampled on small or large spatial scales. Another concern is that our use of wild-caught fish, rather than common-garden fish, could reduce our ability to detect local adaptation. Yet this possibility seems unlikely given that plasticity is often adaptive (Van Kleunen and Fischer 2005; Ghalambor et al. 2007; Auld and Relyea 2011), and so wild-caught individuals would be expected to show even stronger local adaptation than common-garden individuals. Alternatively, oil-polluted environments might plastically reduce the performance of adult guppies even though they are genetically adapted (i.e., counter-gradient variation). In such cases, the use of common-garden fish could enhance the evidence for local adaptation. Although we cannot currently distinguish between these alternatives, our failure to find consistent local adaptation at the phenotypic level does not bode well for finding it at the genetic level. Finally, our interpretation of local (mal) adaptation currently applies only to adults, whereas earlier life stages could be more sensitive to the toxic effects of crude oil (Moles and Rice 1983; Serigstad 1987; Heintz et al. 1999; Bozinovic and Oleksiak 2010; Bozinovic et al. 2013).

A possible biological explanation for the (at best) weak local adaptation is that all of the southern populations we studied—even those in currently not-polluted environments —might have a previous history of oil pollution. This explanation is worth considering given that the entire southern region has a long history (since the early 1900s—an oil seepage would have been common even before that) of spatially and temporally variable oil exploitation (Sutton 1955). Indeed, this possibility was why we also conducted experiments with guppies from northern Trinidad, a region where oil extraction has not been practiced. Given that this second experiment found some evidence of local adaptation, the above idea might have some merit. However, local adaptation was weak and inconsistent even in this second experiment, leading us to the more reasonable interpretation that adaptation to oil pollution is weak in the studied guppy populations, in particular for MR.oil guppies. This low level of local adaptation might stem from a lack of strong divergent selection or from constraints on adaptation. A lack of divergent selection seems unlikely given the observed negative effects of oil on survival and mass change (Fig.6, Fig. S7–S9), which leads us to now more fully consider the constraint hypothesis.

Oil pollution clearly creates stressful conditions and several general explanations exist for why local adaptation might be difficult in such cases (see Introduction). We here use our data to inform some of these possibilities. First, the inbreeding and genetic drift that can result from strong selection (Falk et al. 2012) might limit local adaptation (Blanquart et al. 2012). In our study, heterozygosities were reasonably high (Table S1), but estimated effective population sizes were at levels (43.5–44.3) where drift might counteract selection. Second, gene flow and/or source-sink dynamics could constrain divergent adaptation (Garant et al. 2007), and the essentially zero neutral genetic differentiation and high inferred gene flow within the southern rivers suggest that gene flow is very high between oil-polluted and not-polluted sites (Fig. S5). Thus, genetic drift and gene flow could constrain local adaptation in our study system (Moore et al. 2007): although, interestingly, dramatic and rapid adaptive responses to other selective forces (predation) are repeatedly seen despite low initial numbers of founders, even lower effective population sizes, and minimal (or no) neutral genetic differentiation (Fitzpatrick et al. 2015; Fraser et al. 2015). Given that our assessment of population genetic structure is currently based on a restricted set of microsatellite markers, additional analyses with broad-scale genomic tools would be very valuable.

In addition to drift and gene flow, a particularly important constraint might be the recency and severity (stressfulness) of environmental change. As noted above, the specific locations of oil exploitation—and therefore oil pollution—vary through time and space in southern Trinidad (Sutton 1955; Agard et al. 1993). Thus, the oil-polluted populations we studied might only recently have been affected and thus might not yet have had enough time to adapt. Although guppies can rapidly adapt to altered biotic conditions (O' Steen et al. 2002; Gordon et al. 2009; Fitzpatrick et al. 2015), perhaps adaptation to the stressful abiotic conditions imposed by oil pollution is more difficult. Indeed, many other species seem to have difficulties adapting to stressful environments (Meyer and Di Giulio 2002; Meyer et al. 2002; Ownby et al. 2002; Johnson et al. 2008; Hicken et al. 2011; Clark et al. 2013). In this scenario, the persistence of guppies in oil-polluted habitats might reflect the fact that guppies are generally less sensitive to crude oil than are most other species, which could thereby reduce the competition and predation that otherwise negatively impact guppies. Indeed, guppy populations are known to achieve higher densities in the absence of predators and competitors (Reznick and Endler 1982; Magurran 2005). Moreover, other empirical and theoretical studies have shown that a lack of predators and competitors can allow populations to persist despite poor adaptation to local abiotic conditions (Fleeger et al. 2003; Johnston and Keough 2003; Mayer-Pinto et al. 2010). This seems a fascinating possibility to consider in the particular case of stressful conditions—persistence might not depend so much on a species’ local adaptation to those conditions but rather on the simple convenience of being less maladapted than their enemies and competitors.

### Applied relevance

General discussions of local adaptation tend to emphasize studies that confirm the typical expectations: (i) survival in a given environment is higher for populations from that environment than for populations from alternative environments, and (ii) adaptation of a population to its home environment should reduce its adaptation to alternative environments (Endler 1986; Schluter 2000; Kawecki and Ebert 2004; Hereford 2009). By contrast, we here emphasize that the frequent exceptions to these predictions are no less interesting or important, especially in the context of human-induced disturbances of natural habitats. Anthropogenic impacts (such as pollution) often exert stronger selection than do more ‘natural’ environmental changes (Hendry and Kinnison 1999; Reznick and Ghalambor 2001; Hendry et al. 2008), and it is of great practical interest for conservation biology to understand how and why natural populations succeed or fail to adapt (Vitousek et al. 1997; Palumbi 2001; Stockwell et al. 2003). It is therefore important to not only scrutinize successful local adaptation to anthropogenic impacts but also the lack thereof.

Our study presents one such exception. Despite high guppy abundances in the oil-polluted sites, as well as evidence of some parallel habitat-associated morphological divergence, reciprocal transplant experiments failed to show local adaptation in fitness components (survival and growth) on a small scale and showed only weak and inconsistent adaptation on a large scale. Although negative results such as these are frequently dismissed as resulting from methodological artifacts (e.g., not the right environments, not the right fitness surrogates, not long enough time periods, and not the right life-history stage), it is just as reasonable that they reflect real biological phenomena associated with particular adaptive contexts. One such phenomenon is the difficulty of adapting to stressful conditions—in this case oil pollution—perhaps combined with its relatively recent advent in our study sites. We encourage further exploration of the exceptions to the expected patterns of local adaptation in general, and especially in relation to stressful disturbances. Perhaps the apparent exceptions really reflect a general pattern and can help us to understand how we might aid population persistence even when local adaptation is weak—as we would expect immediately after most environmental changes. For instance, the possibility that local adaptation of focal species becomes less critical when that species’ enemies are even less well adapted, suggests the value of an integrated ‘evolutionary impact assessments’ at the community level.

## Data Archiving Statement

Data available from the Dryad Digital Repository: http://dx.doi.org/10.5061/dryad.n82dm.

## Supporting Information

**Data S1** Supplementary Material. **Figure S1.** Landmarks (LMs) used in this study: (1) lower mouth, (2) tip of snout, (3) inflection point where operculum intersects outline, (4) dorsal fin intersection 1, (5) dorsal fin intersection 2, (6) caudal fin upper intersection with main body, (7) caudal fin middle intersection with main body, (8) caudal fin lower intersection with main body, (9) anal fin intersection 1, (10) anal fin intersection 2, (11) inflection point where operculum intersects outline, (12) intersection where the straight line between the center of the eye and intersection point 7 intersects with the operculum. For the full body shape analyses we used LMs 1-11 (LM set 1) and for the head shape analyses we used LMs 1, 2, 3, 12, and 11 (LM set 2). **Figure S2.** Patterns of local adaptation predicted from three non-mutually exclusive hypotheses tested in the mixed model analyses. **Figure S3.** Evaluation of the number of population clusters across both rivers based on the rate of change in the log probability (Evanno et al. 2005) of microsatellite data in successive STRUCTURE runs for an assumed *k* = 1 – 6 populations. **Figure S4.** Coefficients of admixture for each individual across all populations estimated using STRUCTURE with the implemented locprior model. **Figure S5.** Posterior probability distributions of migration rates between oil-polluted and not-polluted sites within each river estimated using IMa2. **Figure S6.** Mean scores (with error bars depicting standard deviations) for the first canonical variate (CV1) calculated from the MANCOVA models on (A) the first 15 PCs from the GPA on body shape and (B) the first five PCs from the GPA on head shape. MANCOVA models included population (MR.oil, MR.np, VR.oil, VR.np) as the grouping factor and centroid size as a covariate. **Figure S7.** Population-level adaptation in the 2011 field transplant experiments. **Figure S8.** Population-level adaptation in the 2012 laboratory transplant experiments. **Figure S9.** Cox proportional hazard survival functions from laboratory transplant experiments in 2012. **Figure S10.** Distribution of local adaptation measures reproduced from the Hereford (2009) meta-analysis. **Figure S11.** Photographic evidence of the extent of oil pollution due to seepage at field sites VR.oil (1, 3) and MR.oil (2, 4, 5). **Table S1.** Microsatellite marker info for all four studied populations, all P-values were corrected for false discovery rate. **Table S2.** Fixed effect predictors of population-level adaptation (Hypothesis 2, see Methods) in the 2011 field experiments. **Table S3.** Population-specific home-site superiority (Hypothesis 3, see Methods) for fitness surrogates survival and mass change in the 2011 field experiments. **Table S4.** Fixed effect predictors of population-level adaptation (Hypothesis 2, see Methods) in the 2012 laboratory experiments. **Table S5.** Population-specific home-site superiority (Hypothesis 3, see Methods) for fitness surrogates survival and mass change in the 2012 laboratory experiments.
