# Supplementary material for: Do stressful conditions make adaptation difficult? Guppies in the oil-polluted environments of southern Trinidad
Source: Evol Appl. 2015 Sep 4;8(9):854–70. doi: 10.1111/eva.12289 (PMC4610383; doi:10.1111/eva.12289)
Supplement: Supplementary file 1 — Data S1 Supplementary Material. Figure S1. Landmarks (LMs) used in this study: (1) lower mouth, (2) tip of snout, (3) inflection point where operculum intersects outline, (4) dorsal fin intersection 1, (5) dorsal fin intersection 2, (6) caudal fin upper intersection with main body, (7) caudal fin middle intersection with main body, (8) caudal fin lower intersection with main body, (9) anal fin intersection 1, (10) anal fin intersection 2, (11) inflection point where operculum intersects outline, (12) intersection where the straight line between the center of the eye and intersection point 7 intersects with the operculum. For the full body shape analyses we used LMs 1-11 (LM set 1) and for the head shape analyses we used LMs 1, 2, 3, 12, and 11 (LM set 2). Figure S2. Patterns of local adaptation predicted from three non-mutually exclusive hypotheses tested in the mixed model analyses. Figure S3. Evaluation of the number of population clusters across both rivers based on the rate of change in the log probability (Evanno et al. 2005) of microsatellite data in successive STRUCTURE runs for an assumed k = 1 – 6 populations. Figure S4. Coefficients of admixture for each individual across all populations estimated using STRUCTURE with the implemented locprior model. Figure S5. Posterior probability distributions of migration rates between oil-polluted and not-polluted sites within each river estimated using IMa2. Figure S6. Mean scores (with error bars depicting standard deviations) for the first canonical variate (CV1) calculated from the MANCOVA models on (A) the first 15 PCs from the GPA on body shape and (B) the first five PCs from the GPA on head shape. MANCOVA models included population (MR.oil, MR.np, VR.oil, VR.np) as the grouping factor and centroid size as a covariate. Figure S7. Population-level adaptation in the 2011 field transplant experiments. Figure S8. Population-level adaptation in the 2012 laboratory transplant experiments. Figure S9. Cox proportional ha [file eva0008-0854-sd1.docx]

**Do stressful conditions make adaptation difficult?**

**Guppies in the oil-polluted environments of southern Trinidad**

**Supplementary Material**

1. **Petroleum hydrocarbon analyses**

1.1 **SPMD analysis (2011 samples)**

1.2 **TPH analysis (2012 samples)**

1. **Supplementary figures**
2. **Supplementary tables**
3. **Literature**

1. **Petroleum hydrocarbon analyses**

1.1 **SPMD analysis**

**Methods (SPMD analysis)**

Comprehensive two dimensional gas chromatography mass with time-of-flight mass spectrometry (GCxGC-TOF-MS) has proved to be extremely useful for characterisation and identification of toxic hydrocarbons and acids dissolved in waters (e.g. Rowland et al. 2011; Scarlett et al. 2011). This highly sophisticated analysis was applied to better characterise the organic petrogenic compounds that were bioavailable in the streams under investigation.

SPMDs were purchased from Environmental Sampling Technologies (EST, Inc. Saint Joseph, MO, USA). These were deployed for 30 days in locations VR.oil, VR.np, MR.oil, and MR.np together with field blanks using standard protocols (Esteve-Turrillas et al. 2008). Following retrieval the SPMDs were stored frozen (–60 °C) prior to extraction by dialysis into cyclohexane using standard protocols performed by National Laboratory Service (Starcross, UK). Following extraction, gravimetric analysis was performed to quantify total organic extract weights. As fatty acids can sometimes be present, the extracts were derivatized using (99:1) bstfa + tmcs (Sigma, Gillingham, UK) then cleaned on alumina columns (5% deactivated) with increasingly polar solvents (Scarlett et al. 2011). The samples were diluted as necessary and the non-polar and mid-polar eluents analysed by gas chromatography mass spectrometry (GC-MS). Due to the extreme complexity of the extracts from SPMDs deployed in the oil-polluted rivers, a subset of these (MR.oil, VR.oil, VR.np, MR.oil field blank) were further analysed by GCxGC-TOF-MS (Scarlett et al. 2011).

**Clean-up of SPMD extracts**

SPMD extracts were adsorbed onto deactivated alumina (4.5% Milli-Q water w/w) and eluted with increasingly polar solvents, hexane, hexane:dichloromethane (1:1) and dichloromethane (100%). Prior to analysis by GC-MS and GCxGC-MS the extract derived from VR.oil was diluted by a factor of 20.

**GC-MS of SPMD extracts**

For GC-MS, extracts were examined on an Agilent GC-MSD (Agilent Technologies, Wilmington, DE, USA). This comprised a 7890A gas chromatograph fitted with a 7683B Series autosampler and a 5975A quadrupole mass selective detector. The column was a HP-5MS fused silica capillary column (30 m x 0.25 mm internal diameter x 0.25 µm film thickness). The carrier gas was helium at a constant flow of 1.0 mL min ^-1^. A 1.0 µL sample was injected into a 300 °C splitless injector. The oven temperature was programmed from 40 to 300 at 10 °C min ^-1^ and held for 10 min.

**GCxGC-MS of SPMD extracts**

Comprehensive multidimensional gas chromatography–mass spectrometry (GCxGC-MS) analyses were conducted using an Agilent 7890A gas chromatograph (Agilent Technologies, Wilmington, DE) fitted with a Zoex ZX2 GCxGC cryogenic modulator (Houston, TX, USA) interfaced with an Almsco BenchTOFdx™ time-of-flight mass spectrometer (Almsco International, Llantrisant, Wales, UK). The first-dimension column was a 100% dimethyl polysiloxane 60 m x 0.25 mm x 0.25 µm Rxi®-1ms (Restek, Bellefonte, USA), and the second-dimension column was a 50% phenyl polysilphenylene siloxane 2.5 m x 0.1 mm x 0.1 µm BPX50 (SGE, Melbourne, Australia). Helium was used as carrier gas and the flow was kept constant at 1.0 mL min^-1^**.** Samples (1µL) were injected at 300°C splitless. The oven was programmed from 40°C (hold for 1 min), then heated to 130°C at 10°C min^-1^ then at 2°C min^-1^ to 320°C (held for 15 min). The modulation period was 6s. The MS transfer line temperature was 290°C and ion source 300°C. Data processing was conducted using GC Image™ v2.1 (Zoex, Houston, TX, USA).

**Results (SPMD analysis)**

The following graphs (G1 – G13) depict two-dimensional total ion chromatograms illustrating GcxGC chromatographic resolution of bioavailable PAH compounds detected with SPMDs deployed at the respective sites.

**G1. MR.oil sample:** Total ion current (TIC)

Extract contains typical petrogenic hydrocarbons including numerous toxic mono- and polycyclic aromatic hydrocarbons (PAH); i.e. mainly branched alkylated structures.


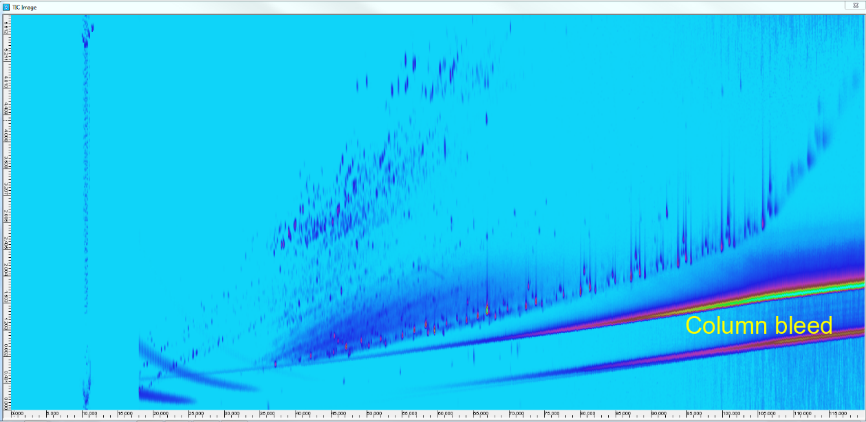


**G2. MR.oil sample:** Branched alkylobenzenes (BABs)


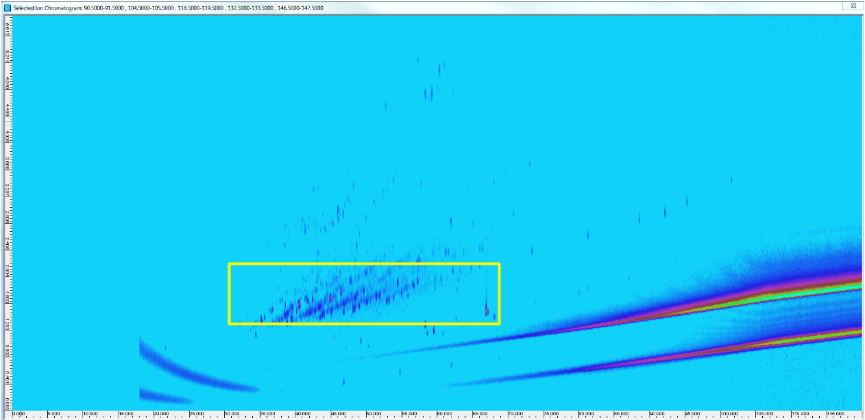


**G3. MR.oil sample:** Branched alkylnaphthalenes (BANs)


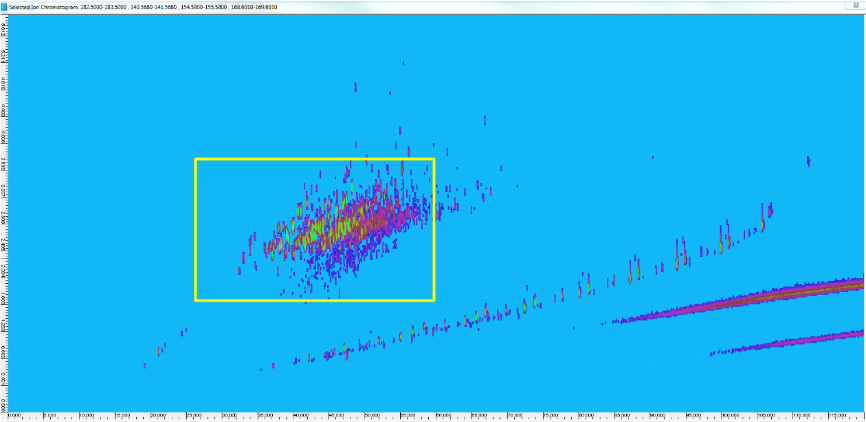


**G4. MR.oil sample:** Phenanthrenes


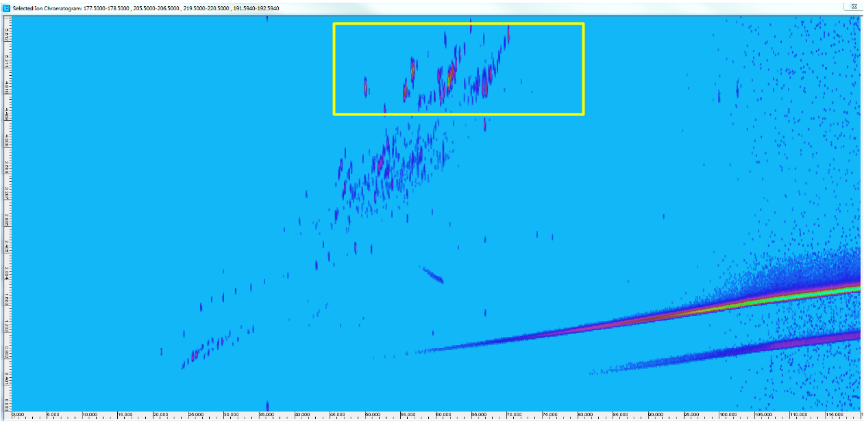


**G5. MR.oil sample:** Dibenzo-Thiophenes


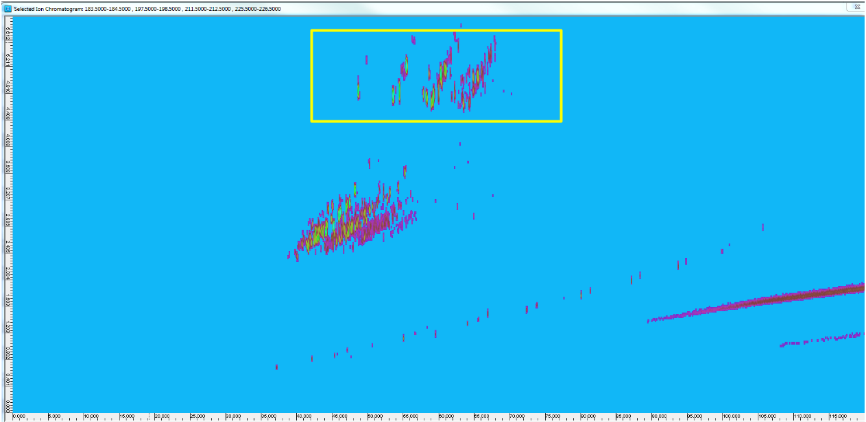


**G6 VR.np sample:** Total ion current (TIC)

Peaks present at very low abundance with ions and retention times consistent with naphthalenes, phenanthrenes, and other PAHs. For comparison: phenanthrene is about 30,000 times lower in total extract of sample VR.oil.


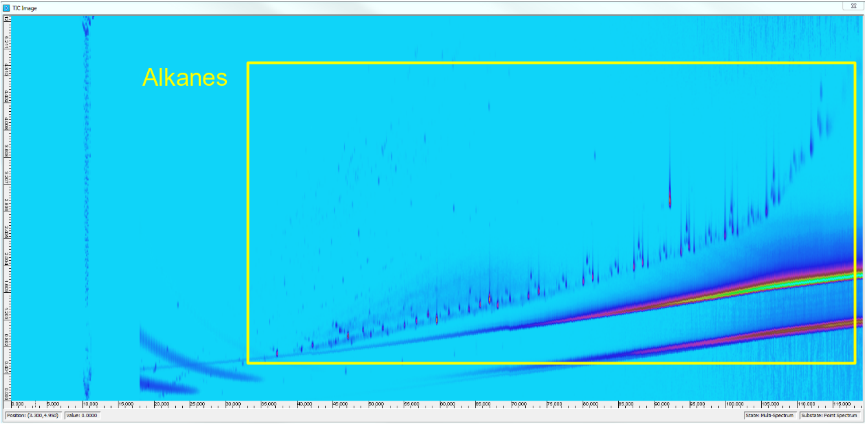


**G7 VR.oil sample:** Total ion current (TIC)

Extract contains typical petrogenic hydrocarbons including numerous toxic mono- and polycyclic aromatic hydrocarbons (PAH); i.e. mainly branched alkylated structures. Note that this sample was 20x diluted compared to others.


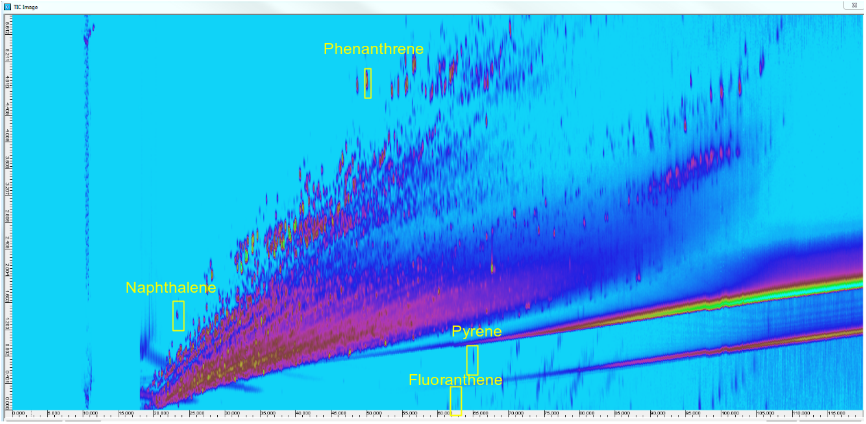


**G8 VR.oil sample:** Branched alkylobenzenes (BABs)


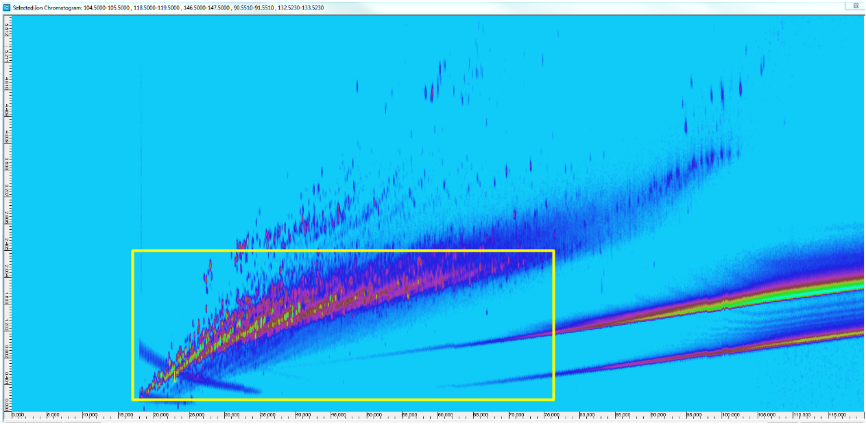


**G9 VR.oil sample:** Branched alkylobenzenes (BANs)


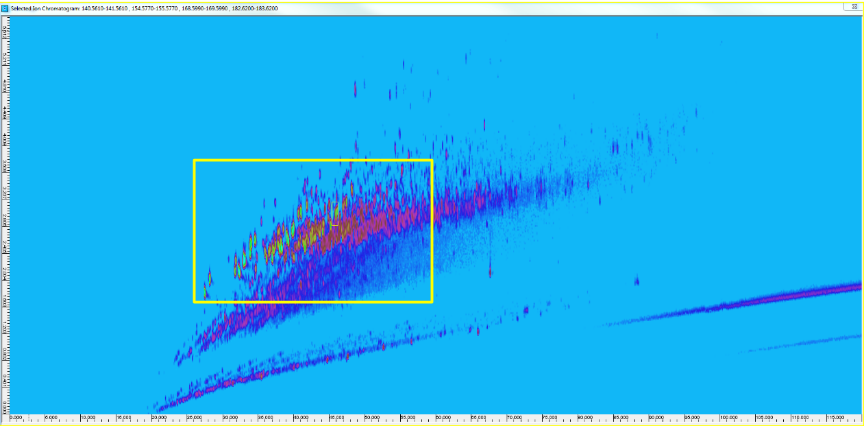


**G10 VR.oil sample:** Phenanthrenes


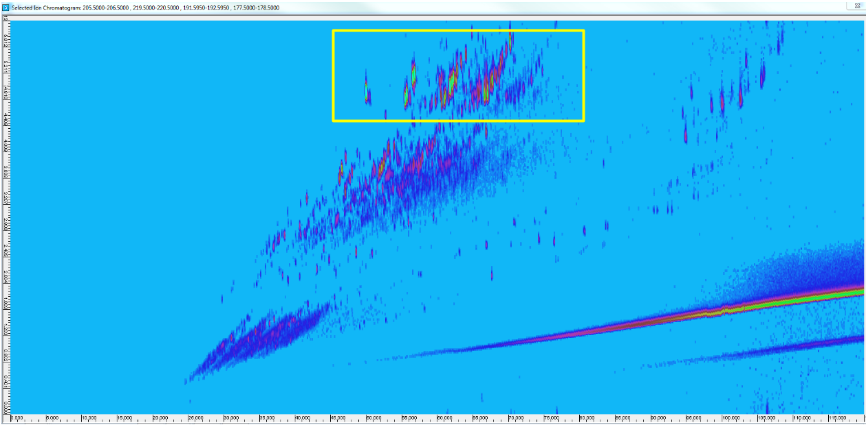


**G11 VR.oil sample:** Dibenzo-Thiophenes


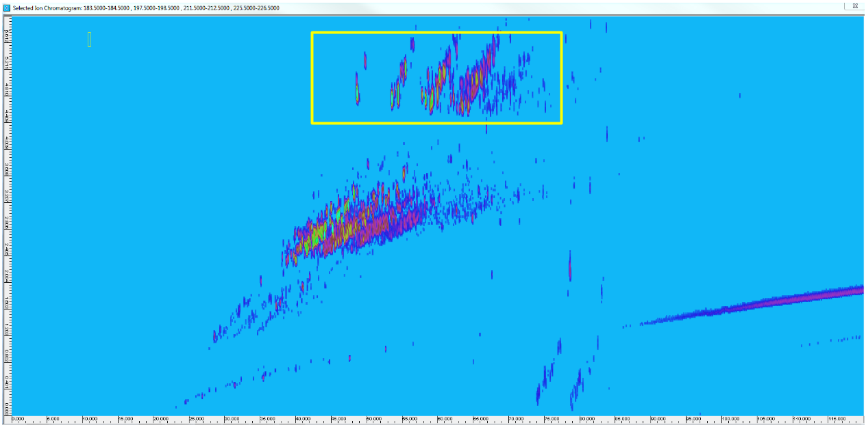


**G12 PAH mixture for both sites:** The graph is 'wrapped' in 2nd dimension to improve separation fro environmental petrogenic sample analysis.


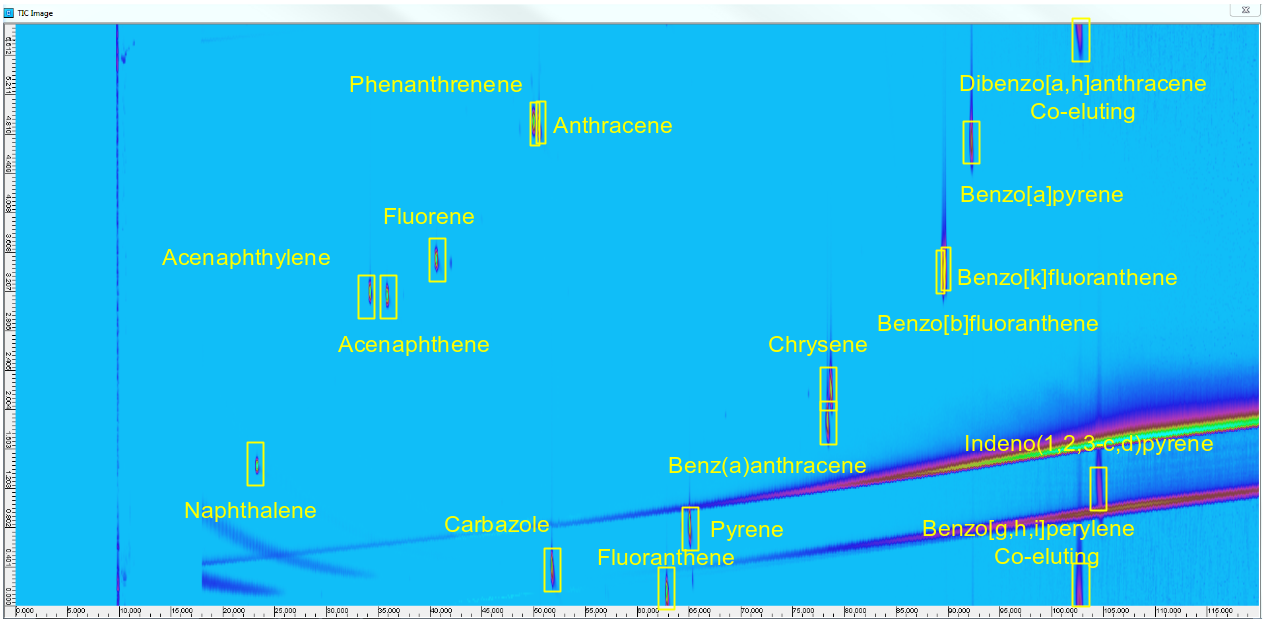


**G13 Field blank MR.oil:** No evidence of PAH or other oil-related toxicants.


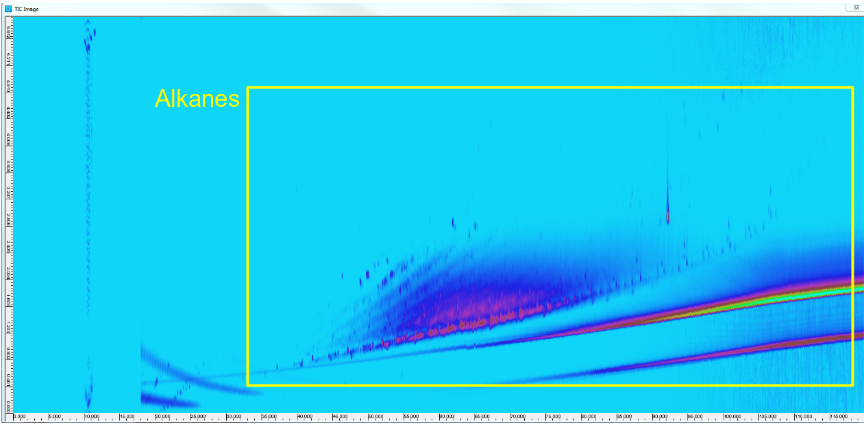


1.2 **TPH analysis**

**Methods (TPH analysis)**

Water samples (~ 400 ml) where collected at two different locations at each of the four field sites (MR.oil, MR.np, VR.oil, and VR.np) and pooled within sites to represent overall pollution levels at the site. The samples were then analyzed through N-Hexane extractable material (EPA 1999) gravimetry analysis at the University of the West Indies, Trinidad. These data revealed that water samples from the two polluted sites (MR.oil and VR.oil) showed strong hydrocarbon contamination, whereas samples from the two clean sites (MR.np and VR.np) both came out below the detection limit (<< 5 mg/L).

Following up on these results, we conducted a more detailed analysis on water samples from the contaminated sites that were stored in our freezer at McGill University since 2012 (the year the study was conducted): The detailed extraction procedure was reported elsewhere (Akbari and Ghoshal 2014). Briefly, the extraction was performed by vigorous mixing of 10 mL of aqueous phase with 40 mL of a mixture of methylene chloride and hexane (50-50% by wt) for 30 min. The organic phase was then separated and passed through a sodium sulphate-silica gel column. The column was then rinsed with methylene chloride-hexane mixture as eluent solvent. The eluent solvent with extracted organic phase was concentrated under nitrogen atmosphere. The sample was then injected to a 6890 Agilent GC with DB1 column. The oven temperature of GC was increased from 40 °C to 280 °C at the rate of 15 °C/min. Helium was used as carrier gas at a flow rate of 10 mL/s. The total petroleum hydrocarbon concentration was determined by integrating the area under peak for C10-C34 hydrocarbons. To track the extraction efficiency, samples were initially spiked with surrogate solution of O-terphenyl in methylene chloride. As another quality control measure, a blank sample of distilled water spiked with surrogate was also analyzed.

**Results (TPH analysis)**

Total petroleum hydrocarbon (TPH) concentration of the VR.oil sample was 214.8+37.87 mg/L based on t analyses of triplicate samples. The chromatogram of the sample is shown in Fig. A. The detected hydrocarbon compounds were primarily high molecular weight, non-volatile petroleum hydrocarbons in the range of C10-C34 (F3 fraction according to CCME standard, (CCME 2001). TPH content of MR.oil sample was also determined as 57.58+8.09 mg/L. As shown in Fig. B, the TPH content was mainly contributed to one prominent peak in the F3 fraction range.

**Figure A**  Chromatogram VR.oil sample

**
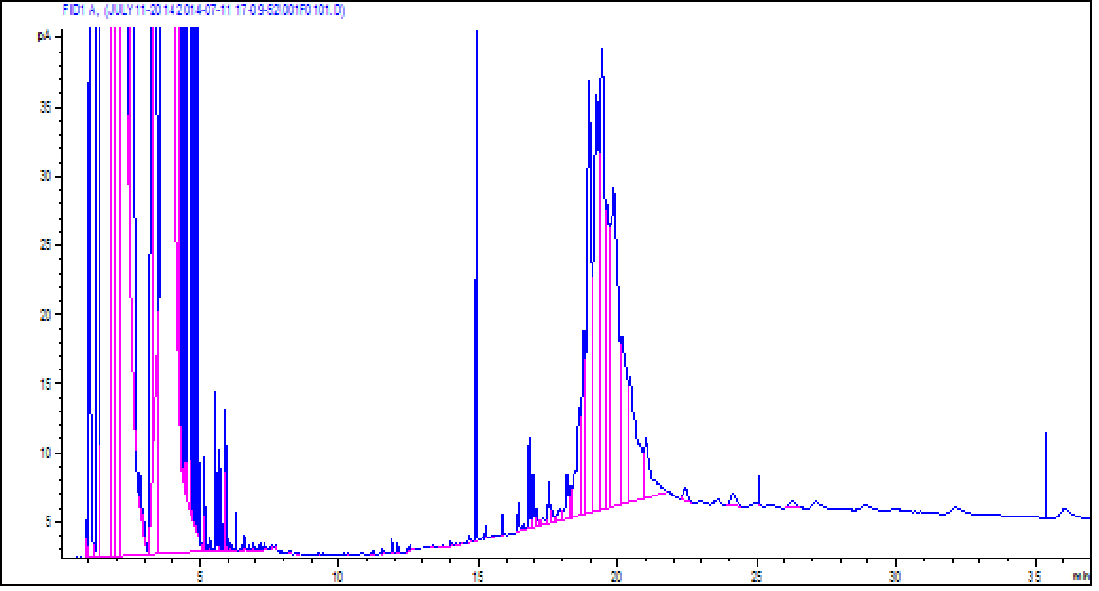
**

**Figure B**  Chromatogram MR.oil sample


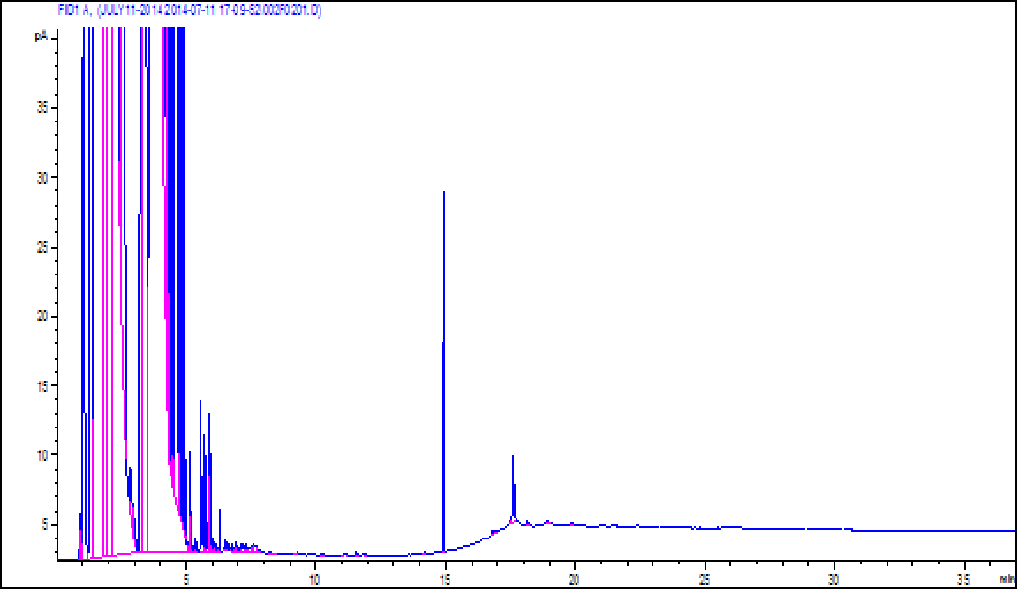


**2. Supplementary figures**

**Figure S1** Landmarks (LMs) used in this study: (1) lower mouth, (2) tip of snout, (3) inflection point where operculum intersects outline, (4) dorsal fin intersection 1, (5) dorsal fin intersection 2, (6) caudal fin upper intersection with main body, (7) caudal fin middle intersection with main body, (8) caudal fin lower intersection with main body, (9) anal fin intersection 1, (10) anal fin intersection 2, (11) inflection point where operculum intersects outline, (12) intersection where the straight line between the center of the eye and intersection point 7 intersects with the operculum. For the full body shape analyses we used LMs 1-11 (LM set 1) and for the head shape analyses we used LMs 1, 2, 3, 12, and 11 (LM set 2).


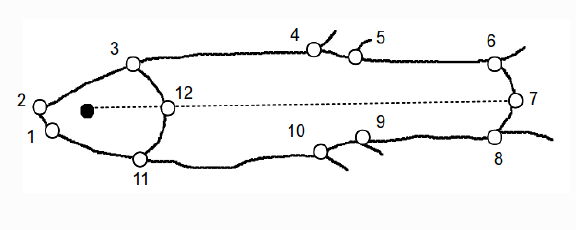


**Figure S2** Patterns of local adaptation predicted from three non-mutually exclusive hypotheses tested in the mixed model analyses. Left panel (Hypothesis 1): “environment-level adaptation” invokes the comparison of similar habitats versus different habitats (▲: populations native to oil-polluted habitats, ●: populations native to not-polluted habitats). Middle panel (Hypothesis 2): “population-level adaptation”, invokes the native (▲) versus non-native (●) comparison by predicting higher fitness for native populations in their home site for all four populations. The distribution of fitness among the three remaining populations can vary according to the test site. Right panel (Hypothesis 3): “population-specific superiority”, predicts population specific superiority for each of the four study populations in their respective home-site (▲) versus the remaining three sites individually.


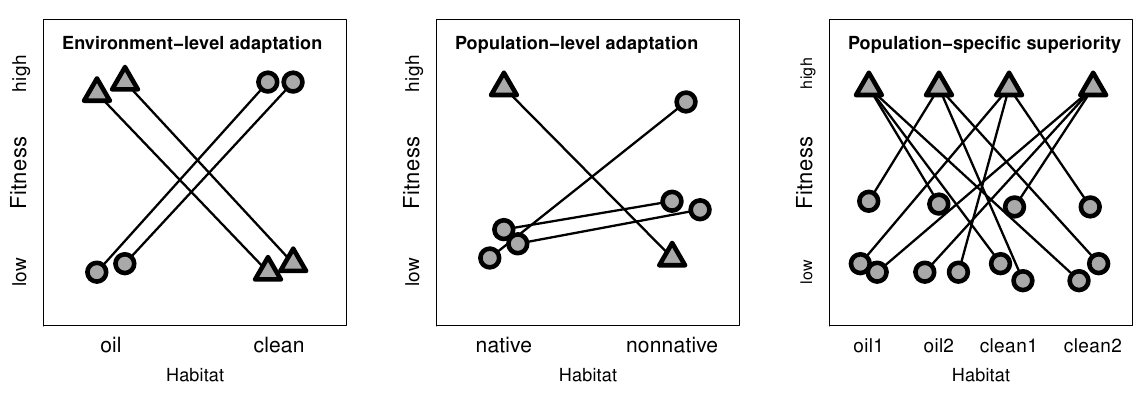


**Figure S3** Evaluation of the number of population clusters across both rivers based on the rate of change in the log probability (Evanno et al. 2005) of microsatellite data in successive STRUCTURE runs for an assumed k = 1 – 6 populations.


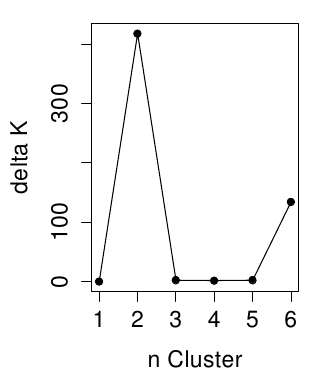


**Figure S4** Coefficients of admixture for each individual across all populations estimated using STRUCTURE with the implemented locprior model. (A) assuming k = 2 populations, and (B) assuming k = 4 populations. Individuals are aligned along the x-axis according to their respective source population.

**(A)**


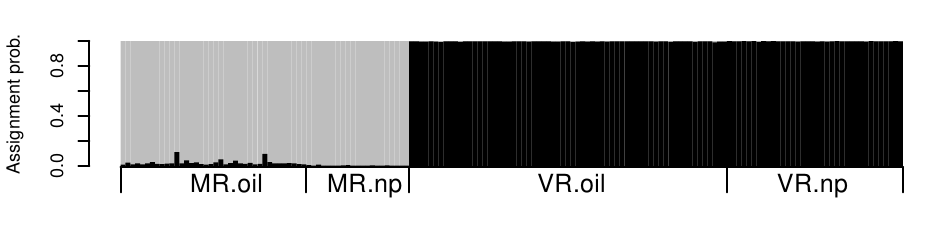


**(B)**


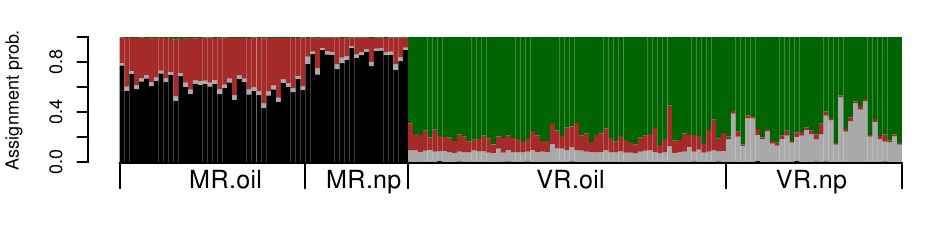


**Figure S5** Posterior probability distributions of migration rates between oil-polluted and not-polluted sites within each river estimated using IMa2. Upper panel: Morne River, lower panel: Vance River.


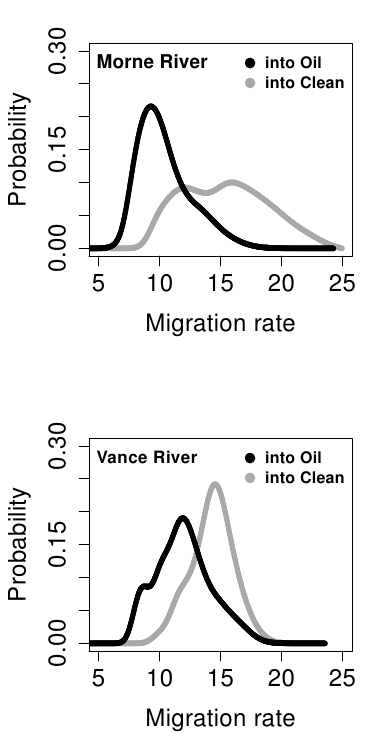


**Figure S6**  Mean scores (with error bars depicting standard deviations) for the first canonical variate (CV1) calculated from the MANCOVA models on (A) the first 15 PCs from the GPA on body shape and (B) the first five PCs from the GPA on head shape. MANCOVA models included population (MR.oil, MR.np, VR.oil, VR.np) as the grouping factor and centroid size as a covariate.


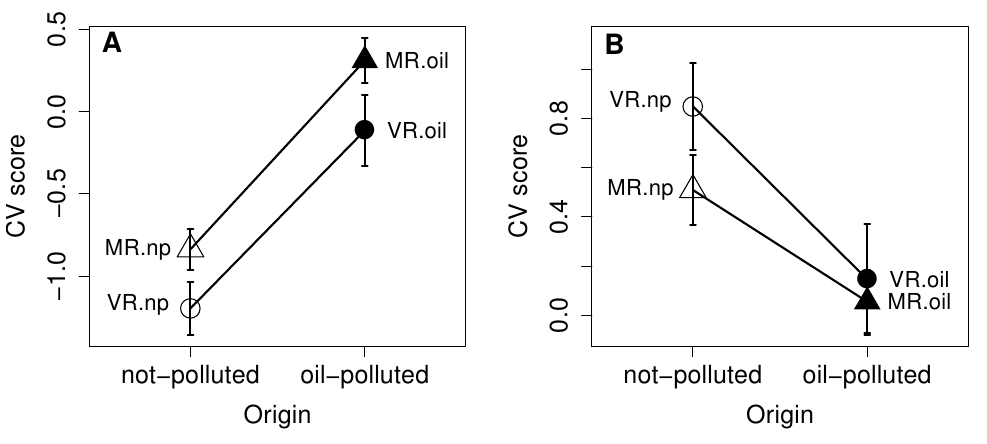


**Figure S7** Population-level adaptation in the 2011 field transplant experiments. Figures depict average population contrasts (+95%CI) between oil-polluted and not-polluted environments for the Morne River (MR.oil and MR.np; upper panel) and the Vance River (VR.oil and VR.np; lower panel) estimated from (G)LMMs for survival (left panels) and mass change (right panels) as fitness surrogates. Grey rectangles depict populations from not-polluted environments (MR.np and VR.np) and black rectangles depict populations from oil-polluted environments (MR.oil and VR.oil). All (G)LMMs incorporated initial mass as a covariate and a random effect structure that accounted for enclosures nested within sites.


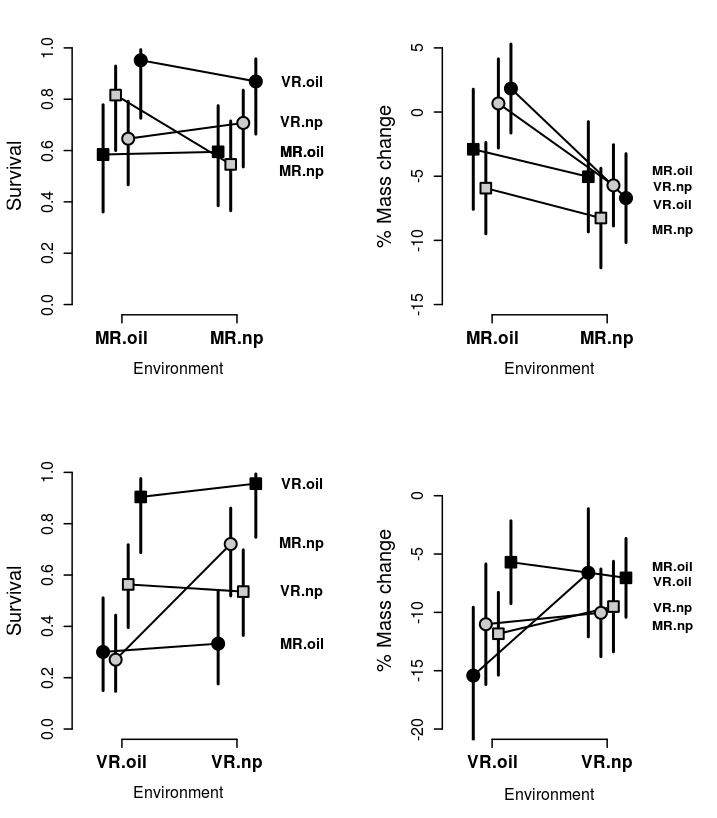


**Figure S8** Population-level adaptation in the 2012 laboratory transplant experiments. Figures depict average population contrasts (+95%CI) in oil-polluted water conditions (MR.oil and VR.oil; upper panel) and not-polluted water conditions (Aripo and Paria; lower panel) estimated from LMMs for survival (left panels) and mass change (right panels) as fitness surrogates. Grey rectangles depict populations from not-polluted environments (Aripo and Paria) and black rectangles depict populations from oil-polluted environments (MR.oil and VR.oil). All (G)LMMs incorporated initial mass as a covariate and a random effect structure that accounted for aquaria nested within blocks.


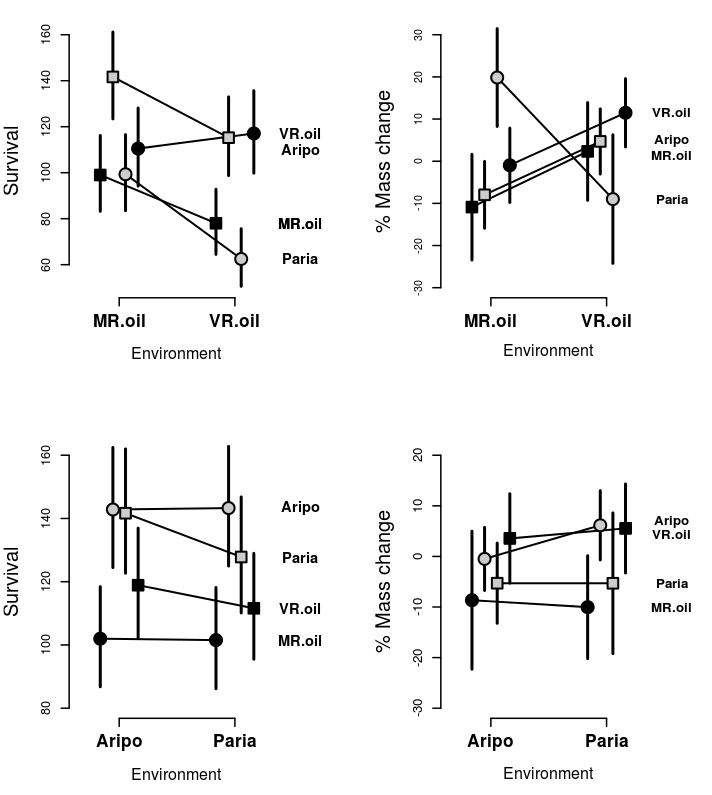


**Figure S9** Cox proportional hazard survival functions from laboratory transplant experiments in 2012. (A) Pooled data, comparing the effect of oil-polluted and clean water conditions across all populations (left panel, “All fish”), only fish from southern Trinidad (VR.oil and MR.oil, “Southern fish”), and fish from northern Trinidad (Paria River and Aripo River, “Northern fish”). (B) Stratified survival functions taking into account the four different water conditions (Ar: Aripo, Pa: Paria, MR.oil: oil-polluted Morne River, VR.oil: oil-polluted Vance River) with each line depicting survival for the respective populations. The gray line depicts 50% survival probability.

**(A)**


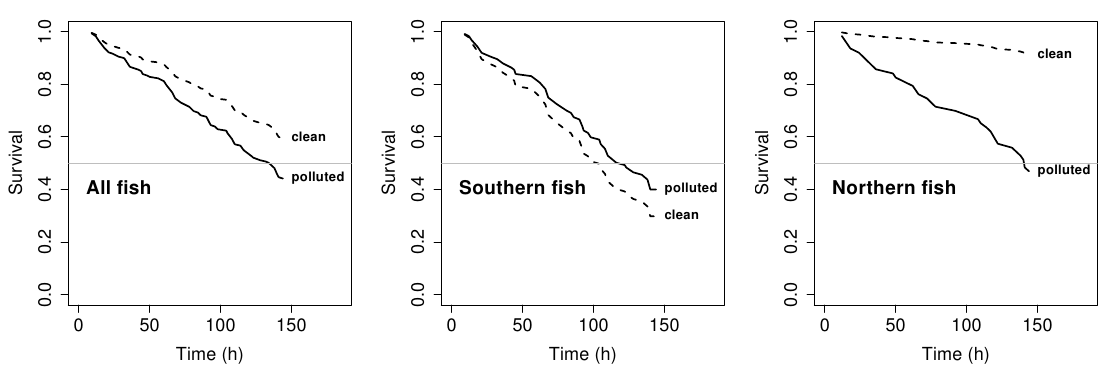


**(B)**


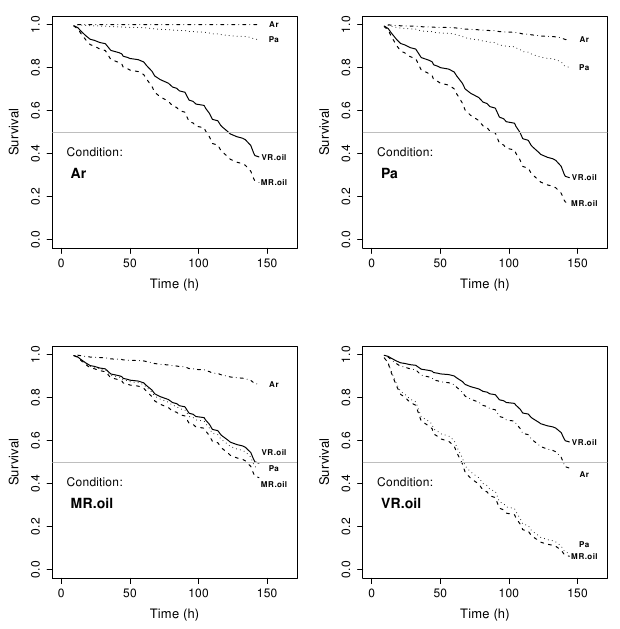


**Figure S10** Distribution of local adaptation measures reproduced from the Hereford (2009) meta-analysis. The red line indicates the overall analysis mean (0.45). Black crosses depict local adaptation measures (relative to the mean fitness at each site) for our study populations in 2011 and 2012.


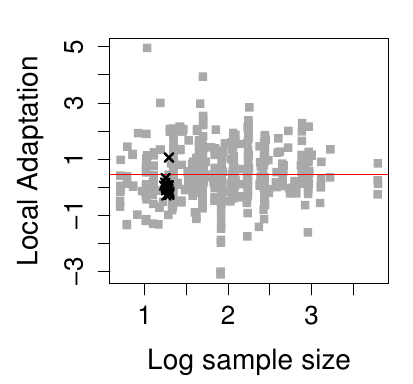


**Figure S11** Photographic evidence of the extent of oil pollution due to seepage at field sites VR.oil (1, 3) and MR.oil (2, 4, 5).


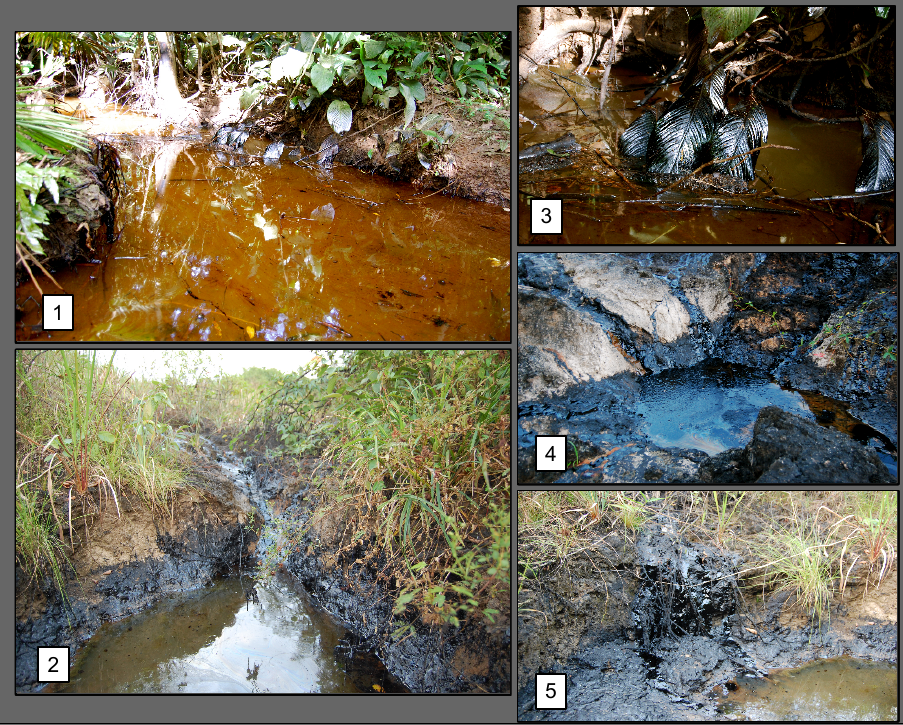


**3. Supplementary Tables**

**Table S1** Microsatellite marker info for all four studied populations, all P-values were corrected for false discovery rate. N depicts the number of alleles and the * refers to a significant deviation from HWE.

**Table S2** Fixed effect predictors of population-level adaptation (Hypothesis 2, see Methods) in the 2011 field experiments. Values depict ANOVA summary statistics of separate (G)LMMs for fitness surrogates survival and mass change. All models included initial mass as a covariate and a random effect structure that accounted for enclosures nested within sites.

**Table S3** Population-specific home-site superiority (Hypothesis 3, see Methods) for fitness surrogates survival and mass change in the 2011 field experiments. Values depict ANOVA summary statistics of separate (G)LMMs in which the contrast for each population's performance in it's native site versus the remaining three sites was represented as fixed effect predictor, and the random effect structure accounted for enclosures nested within sites.

**Table S4** Fixed effect predictors of population-level adaptation (Hypothesis 2, see Methods) in the 2012 laboratory experiments. Values depict ANOVA summary statistics of separate LMMs for fitness surrogates survival and mass change. All models included initial mass as a covariate and a random effect structure that accounted for aquaria nested within blocks.

**Table S5** Population-specific home-site superiority (Hypothesis 3, see Methods) for fitness surrogates survival and mass change in the 2012 laboratory experiments. Values depict ANOVA summary statistics of separate LMMs in which the contrast for each population's performance in it's native site versus the remaining three sites was represented as fixed effect predictor, and the random effect structure accounted for aquaria nested within blocks.

**4. Literature**

Akbari

, A., and S. Ghoshal. 2014. Pilot scale study of bioremediation of a petroleum

hydrocarbon-contaminated clayey soil from a sub-arctic site.

Journal of Hazardous Material **280**:595–602.

CCME. 2001. Reference Method for the Canada-wide Standard for Petroleum Hydrocarbons

in Soil – Tier 1 Method. Canadian Council of Ministers of the Environment, Winnipeg,

MB, Canada.

EPA. 1999. United States Environmental Protection Agency; Method 1664, Revision A: N-

Hexane Extractable Material (HEM; Oil and Grease). Engineering and Analysis Division,

Washington, D.C.

Esteve-Turrillas, F. A., V. Yusa, A. Pastor, and M. de la Guardia. 2008. New perspectives in the use of semipermeable membrane devices as passive samplers. Talanta **74**:443-457.

Rowland, S. J., A. Scarlett, C. West, D. Jones, and R. Frank. 2011. Diamonds in the rough: identification of individual naphthenic acids in oil sands process water. Environmental Science and Technology **45**:3154-3159.

Scarlett, A. G., R. Clough, C. West, C. A. Lewis, A. M. Booth, and S. J. Rowland. 2011. Alkylnaphthalenes: Priority Pollutants or minor contributors to the poor health of marine mussels? Environmental Science and Technology **45**:6160-6166.

Sutton, P. A., C. A. Lewis, and S. J. Rowland. 2005. Isolation of individual hydrocarbons from the unresolved complex hydrocarbon mixture of a biodegraded crude oil using preparative capillary gas chromatography. Organic Geochemistry **36**:963-970.
